# Metalloimmunotherapies: metal-driven regulatory networks in the immune microenvironment

**DOI:** 10.3389/fimmu.2026.1901186

**Published:** 2026-09-16

**Authors:** Bo Wen, Yifei Wu, Xinqi Lian, Xiaoming Li, Benhua Xue, Jimin Guo

**Affiliations:** 1 College of Materials Science and Engineering, Beijing University of Chemical Technology, Beijing, China; 2 College of Life Science and Technology, Beijing University of Chemical Technology, Beijing, China; 3 State Key Lab Organ Inorgan Composites, Beijing University of Chemical Technology, Beijing, China; 4 Beijing Advanced Innovation Center for Soft Matter Science and Engineering, Beijing University of Chemical Technology, Beijing, China; 5 College Information Science & Technology, Beijing University of Chemical Technology, Beijing, China

**Keywords:** immune microenvironment, inflammation, metalloimmunotherapies, metals, signaling pathways

## Abstract

Metal ions, as common endogenous signaling molecules, play a crucial role in immune homeostasis and inflammation regulation. This paper systematically elucidates the molecular mechanisms by which iron, manganese, zinc, copper, magnesium, cobalt, nickel, lithium, silver, gold, and rare earth elements cerium, yttrium and lanthanum regulate inflammation through NF-κB, MAPK, AKT, STAT, and ROS pathways. It proposes establishing a closed-loop “metal-immunotherapeutic efficacy” database to lay the theoretical foundation for the development of metal immune-materials genomics.

## 1 Introduction

Metals have found extensive applications in the medical field. The documented therapeutic use of metals in humans dates back as far as the origins of civilization. As early as ancient India and the Roman Empire, copper was employed to disinfect water and prevent the spread of disease (1). In 1827, American surgeon John Kearney-Rogers first reported the use of silver wire. Systematic research into metallurgy in implants began with H.S. Levert’s work in 1829. He studied gold, silver, lead, and platinum *in vivo* to explore their biocompatibility (2).

In recent years, metal implants have gained widespread application in fields such as orthopedics and dentistry due to their excellent biocompatibility and mechanical properties, which enable them to integrate well with human tissues. Titanium alloys and magnesium alloys, characterized by their low elastic modulus, high strength, and superior corrosion resistance, are extensively used in orthopedic implants (3). Magnesium-based implants can activate the PI3K-Akt pathway through sustained release of Mg²^+^. Zirconium, tantalum, and niobium exhibit excellent high flexural strength and high fracture toughness, but present issues with mechanical stability and local cytotoxicity (4). Cobalt-based alloys exhibit excellent wear resistance, but ion leaching can induce macrophage inflammatory responses (5).

The transition from traditional mineral medicine to modern metallic drugs represents a significant milestone in pharmacology, with the discovery of cisplatin marking the inception of modern metallopharmacology (1). Cisplatin induces single-strand or double-strand DNA breaks in glioma cells, blocking replication and transcription while activating the ATM/ATR pathway to cause cell cycle arrest. If damage is excessive, mitochondrial and oxidative stress amplify the signal, triggering apoptosis. Its cytotoxicity is negatively correlated with DNA double-strand break (DSB) repair capacity; inhibiting repair enhances therapeutic efficacy (6, 7). Traditional platinum-based drugs enhance immunogenicity and suppress tumor immune escape by inducing DNA damage, activating dendritic cells and CD8^+^ T cells, and blocking the PD-1/PD-L1 pathway (8). In contrast, transition metal ions such as Fe³^+^ and Mn²^+^ not only participate in immune regulation and oxidative stress responses, but also possess the ability to directly induce immunogenic cell death (ICD) and activate key immune pathways (such as cGAS-STING) (9). Transition metal (Fe²^+^/Fe³^+^, Cu^+^/Cu²^+^, Zn²^+^) chelators, pre-chelators, and ion carriers can serve as small-molecule cancer chemotherapeutic agents. They have been successfully demonstrated to exhibit cytotoxicity and targeted activity against drug-resistant cancer cells.

Metalloimmunotherapies represent an emerging class of therapeutic strategies that harness or modulate the intricate interactions between metal ions (or metal-ion-containing substances) and the host immune system to regulate physiological or pathological immune responses for disease treatment (10). Distinct from traditional metallopharmacology, which primarily exploits the direct cytotoxicity of coordination complexes to induce DNA damage and cell apoptosis (as exemplified by cisplatin), metalloimmunotherapies pivot toward reshaping the immune ecosystem (11). Grounded in the principles of metalloimmunology, metal ions are recognized not merely as structural elements, but as critical endogenous signaling molecules and catalytic cofactors that actively orchestrate immune homeostasis and inflammation regulation. Understanding the nano-bio interactions between metal-based nanomaterials and immune cells, particularly the mononuclear phagocyte system, is fundamental for predicting their immunological fate and safety profiles (10, 12).

So far, despite extensive research on metals, a systematic summary of how they influence signaling pathways remains incomplete. Conceptually, this manuscript evaluates five major signaling and regulatory networks—NF-κB, MAPK, STAT, AKT, and ROS/oxidative stress—as parallel, mutually interactive pillars of metal-driven immune regulation. While these five cascades carry equal analytical weight in our mechanistic framework, their detailed narrative lengths across sections naturally reflect the current volume of published literature, wherein the NF-κB axis remains the most extensively documented target. It should be noted that rather than focusing on narrow, cell-type-specific immune microenvironments, this review aims to construct a generalized molecular framework. The highlighted elements in the periodic table are the metal elements discussed in this review (**Figure 1**).

**Figure 1:**
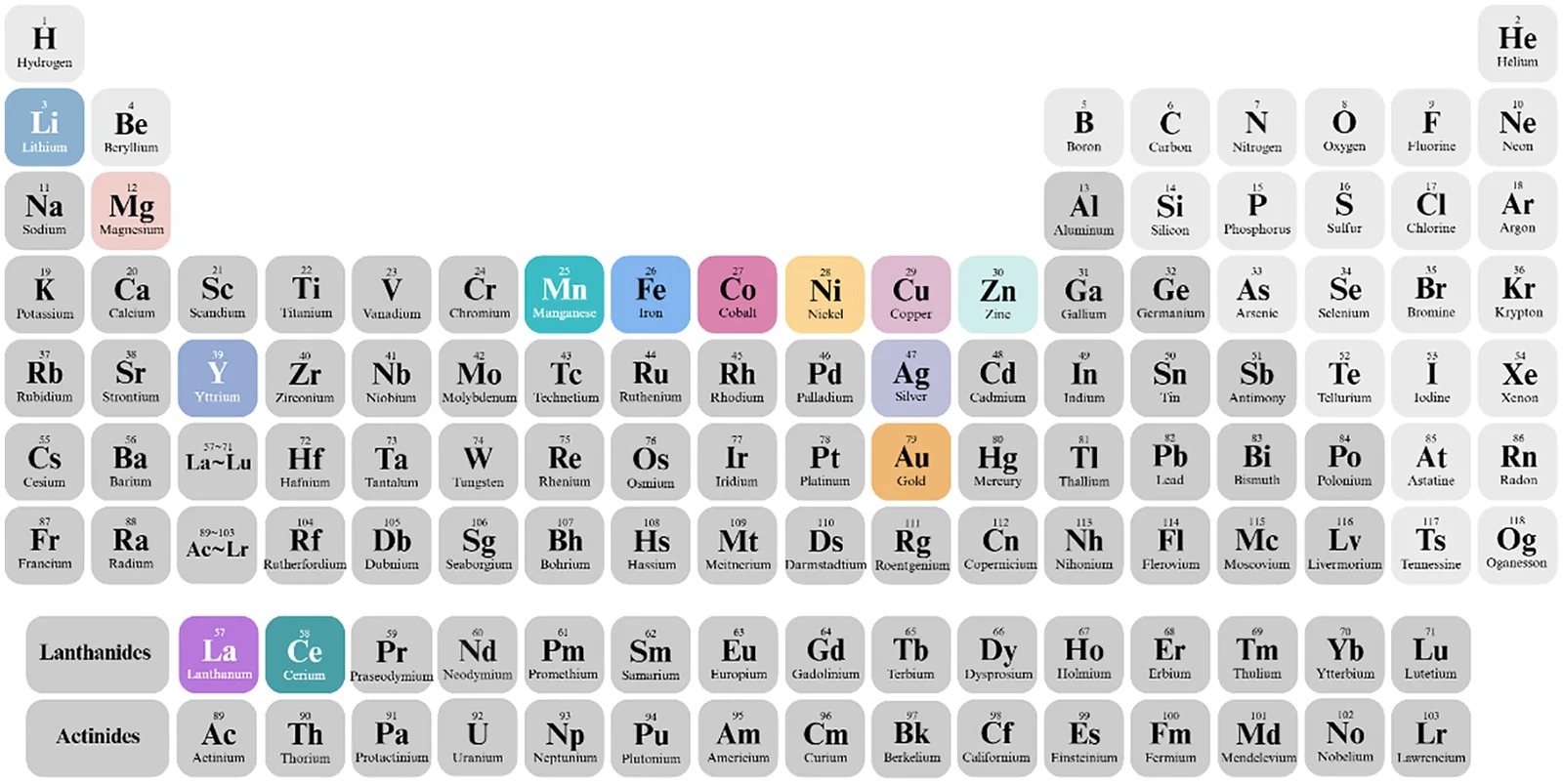
Periodic table: metal ions highlighted are discussed in this paper.

## 2 Pathway affected by metal

### 2.1 NF-κB pathway

The NF-κB (Nuclear Factor κB) signaling pathway serves as a central transcription factor pathway regulating immune and inflammatory responses, mediating rapid cellular reactions to external stimuli. It comprises NF-κB family proteins, including p65/RelA, and p50, which typically remain inactive through binding to the inhibitory protein IκB (13).

External stimuli such as pathogen-associated molecular patterns (PAMPs) activate IκB kinase (IKK) via Toll-like receptors (TLRs), triggering phosphorylation and degradation of IκB. This releases NF-κB, enabling its translocation to the nucleus and subsequent induction of proinflammatory cytokine expression, including TNF-α (Tumor Necrosis Factor Alpha), IL-6 (Interleukin-6), and IL-1β (Interleukin-1 Beta), thereby mediating inflammatory and immune responses (14). These cytokines function as signaling molecules to further regulate immune cell activity, while exposure to metallic elements can modulate inflammatory progression by altering NF-κB pathway activity. The regulatory roles of various metals in the NF-κB pathway are summarized in **Figures 2**, **3**.

**Figure 2:**
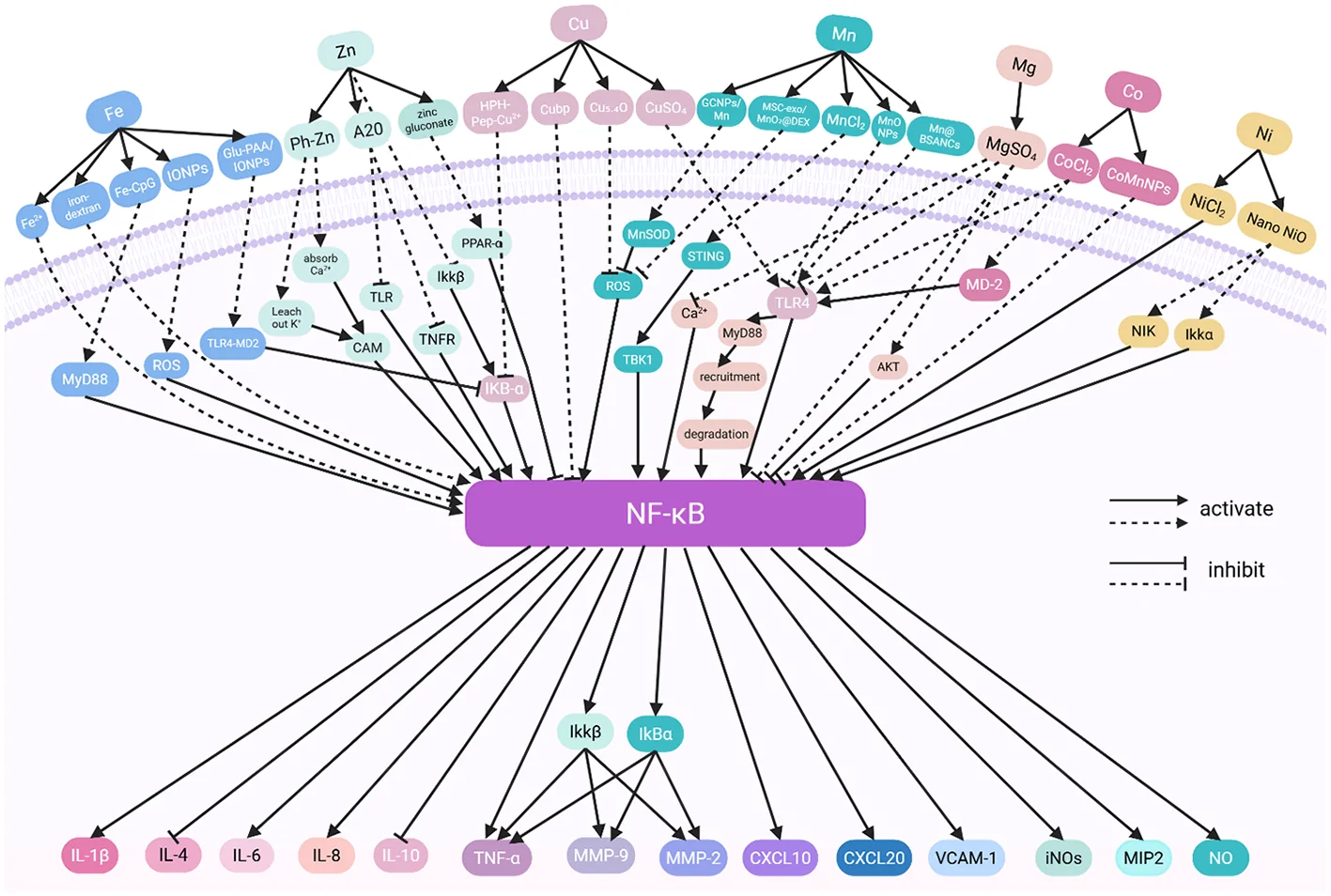
NF-κB pathway regulated by Fe, Zn, Cu, Mn, Mg, Co, and Ni. NF-κB, Nuclear Factor Kappa B; IKK, IκB Kinase; IκBα, Inhibitor of Nuclear Factor Kappa B Alpha; NEMO, NF-κB Essential Modulator; TLR4, Toll-Like Receptor 4; TNFR, Tumor Necrosis Factor Receptor; IL-1R, Interleukin-1 Receptor; MyD88, Myeloid Differentiation Primary Response Gene 88; IRAK, Interleukin-1 Receptor-Associated Kinase; TRAF6, TNF Receptor-Associated Factor 6; LPS, Lipopolysaccharide; TNF-α, Tumor Necrosis Factor Alpha; IL-1β, Interleukin-1 Beta; IL-6, Interleukin-6; iNOS, Inducible Nitric Oxide Synthase; COX-2, Cyclooxygenase-2; ROS, Reactive Oxygen Species.

**Figure 3:**
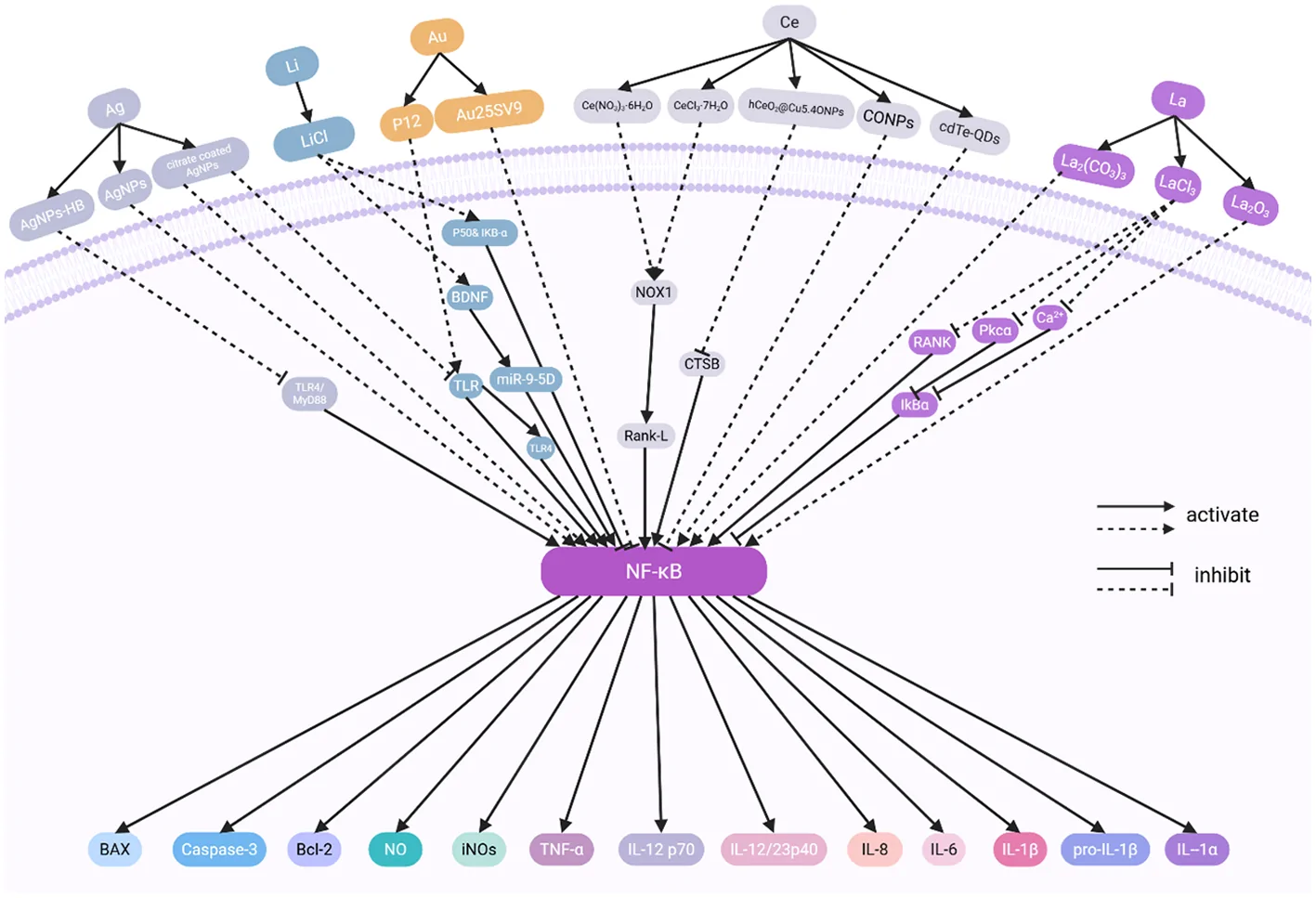
NF-κB pathway regulated by Ag, Li, Au, Ce and La. NF-κB, Nuclear Factor Kappa B; IKK, IκB Kinase; IκBα, Inhibitor of Nuclear Factor Kappa B Alpha; TLR4, Toll-Like Receptor 4; MyD88, Myeloid Differentiation Primary Response Gene 88; TRAF6, TNF Receptor-Associated Factor 6; TNF-α, Tumor Necrosis Factor Alpha; IL-1β, Interleukin-1 Beta; IL-6, Interleukin-6; iNOS, Inducible Nitric Oxide Synthase; COX-2, Cyclooxygenase-2; ROS, Reactive Oxygen Species.

#### 2.1.1 NF-κB pathway affected by iron

##### 2.1.1.1 NF-κB pathway affected by iron ion

Elevated intracellular free ferrous iron (Fe²^+^) activates the NF-κB signaling pathway through enhanced p65 phosphorylation, upregulating proinflammatory factors such as TNF-α, IL-1β, and IL-6 in a rat model of chronic cerebral ischemia. This exacerbates the inflammatory response and promotes ferroptosis (15). In Ward**’**s study (16), iron overload induced by iron dextran, a complex formed by chelation of Fe³^+^ with dextran, significantly enhanced basal NF-κB activity in alveolar macrophages in both pregnancy and chronic iron overload models. Xia et al. demonstrated that marginal iron overload during pregnancy in rats promotes TLR4, MyD88, and NF-κB expression, enhances NF-κB phosphorylation (p-p65), and activates the NF-κB signaling pathway, thereby increasing production of proinflammatory cytokines TNF-α, IL-6, and IL-1β and exacerbating colonic inflammation (17).

##### 2.1.1.2 NF-κB pathway affected by iron-based therapeutic strategies

For iron oxide nanoparticles (IONPs), Yang et al. (18) reported that spherical nucleic acid Fe-CpG constructs, assembled from IONPs, successfully enter cells via endocytosis. In a SARS-CoV-2 subunit vaccine model established in C57BL/6 mice, these constructs directly bind to TLR7/8 and subsequently activate the downstream NF-κB pathway through MyD88. This cascade promotes the robust release of proinflammatory factors, such as TNF-α and IL-23, whose proinflammatory effects ultimately act as a potent adjuvant to enhance the vaccine’s overall immune response.

Beyond infectious disease prophylaxis, IONPs play a pivotal role in modulating antitumor immunity through specific biochemical intracellular networks. Chen et al. (19) revealed that IONPs can generate reactive oxygen species (ROS) via the intracellular Fenton reaction in mouse tumor models, including melanoma and colorectal cancer, thereby triggering the activation of the NF-κB pathway. Concurrently, these nanoparticles exhibit a powerful synergy with the small-molecule STING agonist MSA-2, further amplifying both NF-κB-mediated proinflammatory signaling and STING activation. This dual activation significantly enhances the secretion of key proinflammatory cytokines, such as TNF-α and IL-6, which effectively promotes dendritic cell maturation and antigen cross-presentation, suppresses tumor cell proliferation, and assists tumor vaccines in amplifying therapeutic immune responses.

In the context of direct tumor immunotherapy, targeted surface modification of IONPs has been shown to precisely orchestrate the tumor microenvironment. Liu et al. (20) demonstrated that glucose-modified iron oxide nanoparticles (Glu-PAA/IONPs) specifically bind to the TLR4-MD2 complex on macrophage surfaces in lung cancer, breast cancer, and melanoma mouse models. This receptor binding promotes the phosphorylation and nuclear translocation of the NF-κB p65 subunit, while accelerating IκB-α degradation to fully activate downstream NF-κB signaling pathways. Consequently, tumor-associated macrophages (TAMs) are successfully reprogrammed toward an antitumor M1 phenotype, releasing critical proinflammatory factors such as TNF-α, IL-1β, and IL-6, thereby exerting robust tumor-suppressive therapeutic effects.

#### 2.1.2 NF-κB pathway affected by manganese

##### 2.1.2.1 NF-κB pathway affected by manganese ion

Mn²^+^ activates the cGAS (cyclic guanosine monophosphate-adenosine monophosphate synthase)-STING (stimulator of interferon genes) pathway, promoting STING phosphorylation, which in turn activates downstream TBK1 (TANK-binding kinase 1). Phosphorylated TBK1 then phosphorylates the p65 protein in the NF-κB pathway. Phosphorylated p65 undergoes nuclear translocation, significantly elevating levels of proinflammatory factors such as TNF-α, IL-6, IL-1β, and IL-12 while simultaneously reducing expression of the anti-inflammatory factor IL-10. This reprograms M2 macrophages into M1 macrophages, enhancing their phagocytic and bactericidal capabilities, strengthening antimicrobial and immune activation functions, and thereby participating in the regulation of inflammatory responses and clearance of infections (21, 22). Huang et al. (23) observed that upon binding to TLR4 on macrophage membranes, Mn@BSANCs activate myeloid differentiation factor 88 (MyD88) and TIR-containing IFN-β-inducing adaptor (TRIF). MyD88 further activates the NF-κB pathway, promoting NF-κB phosphorylation (p-NF-κB), while TRIF activates interferon regulatory factor 7 (IRF7). Activated NF-κB translocates to the nucleus, upregulating gene expression and protein secretion of inflammatory mediators such as TNF-α, IL-6, IL-1β, and iNOS (inducible nitric oxide synthase), as well as chemokines CCL2 and CXCL10, thereby promoting inflammatory responses.

##### 2.1.2.2 NF-κB pathway affected by manganese-based therapeutic strategies

For MnOx nanoparticles, MnOx nanoparticles could reduce reactive oxygen species (ROS) accumulation, suppress inflammation-induced responses by clearing pro-inflammatory molecules, blocking TLR9/TLR3/TLR4 activation, thereby inhibiting NF-κB signaling pathway activation (24). According to Tian’s research (25), MSC-exo/MnO_2_@DEX scavenges ROS and generates oxygen, downregulates NF-κB-p65, inhibits IκBα phosphorylation, and blocks NF-κB nuclear translocation. In CCl_4_-induced acute liver injury (ALI) mice, it reduces serum TNF-α and IL-6 levels while increasing IL-10, significantly alleviating hepatic inflammation. Wang et al. (26) revealed that manganese-loaded glucosyl carbon nanoparticles (GCNPs/Mn) release manganese ions via lysosomes, restoring manganese superoxide dismutase (MnSOD) activity impaired in an autoinflammatory vasculitis mouse model (AAV). This reduces reactive oxygen species (ROS) accumulation, thereby inhibiting NF-κB pathway activation, mitigating NET-induced ferroptosis in glomerular endothelial cells (GEnCs) and vascular endothelial injury, and ultimately alleviating renal injury and vasculitis symptoms in mice.

#### 2.1.3 NF-κB pathway affected by zinc

Elevated Zn²^+^ levels inhibit IKKβ, reduce phosphorylation and degradation of IκBα, suppress NF-κB pathway activation, and decrease IL-6 expression in a polymicrobial sepsis mouse model. Zn²^+^ influx from the extracellular space or vesicles into the cytoplasm is mediated by ZIP (zinc-iron transporter) proteins, whereas ZNT (zinc transporter) proteins facilitate reverse export of Zn²^+^ to the extracellular space or vesicles. Metallothionein (MT) buffers intracellular free zinc concentrations through reversible Zn²^+^ binding, cooperating with ZNT/ZIP to regulate zinc homeostasis. This equilibrium is critical for maintaining antioxidant enzyme activity and cellular oxidative defense systems (27). ZIP8 enhances zinc uptake from the extracellular environment or release from intracellular organelles, thereby increasing cytoplasmic zinc levels and acting as a regulator of the NF-κB negative feedback system (28). Zinc ions in zinc gluconate can inhibit NF-κB pathway activation by inducing A20 and PPAR-α (peroxisome proliferator-activated receptor alpha) expression, thereby suppressing TNF-α, IL-1β, and VCAM-1 (vascular cell adhesion molecule 1) expression. Separately, zinc gluconate has also been reported to reduce the lipid peroxidation products MDA (malondialdehyde) and HAE (hydroxyalkenals), together attenuating oxidative and inflammatory responses (29, 30).

Jarosz et al (31) also identified zinc finger protein A20, also known as tumor necrosis factor alpha-induced protein 3 (TNFAIP3), as an effective anti-inflammatory protein that limits the duration and intensity of signaling for several proteins involved in the NF-κB pathway. In LPS- and TNF-α-induced inflammatory responses, A20 binds zinc to become activated, thereby inhibiting TLR- and TNFR-mediated NF-κB pathway activation. This reduces IL-1β and TNF-α gene expression, ultimately suppressing the inflammatory response.Yan et al. (32) demonstrated that zinc sulfate (ZnSO_4_) inhibits the classical NF-κB signaling pathway in a calcium chloride (CaCl_2_)-induced rat abdominal aortic aneurysm (AAA) model by inducing zinc finger protein A20. This downregulates NF-κB p65 subunit expression, reduces phosphorylation of IKKβ and IκBα, prevents IκBα degradation, downregulates MMP-2 (matrix metalloproteinase-2) and MMP-9 (matrix metalloproteinase-9), and diminishes TNF-α-induced inflammatory responses.

Additionally, Tan (33) confirmed that Zn²^+^ from Ph-Zn nanosheets binds to the potassium channel Kv1.3 in mouse breast cancer (4T1 cell line) and colorectal cancer (CT26 cell line) models, leading to K^+^ efflux and Ca²^+^ influx. This activates calmodulin (CaM), which in turn activates the NF-κB pathway, promoting p65 expression and enhancing IL-1β secretion.

#### 2.1.4 NF-κB pathway affected by copper

##### 2.1.4.1 NF-κB pathway affected by copper ion

Copper ions induce oxidative stress and catalyze the generation of reactive oxygen species (ROS), thereby activating TLR4 in a rat model of acute cardiac toxicity induced by copper sulfate (CuSO_4_). Following TLR4 activation, the MyD88 pathway recruits and activates IRAK (interleukin-1 receptor-associated kinase), TRAF6 (tumor necrosis factor receptor-associated factor 6), and TAK1 (transforming growth factor β-activated kinase 1). TAK1 phosphorylates the IKK complex, which in turn phosphorylates IκBα, leading to its degradation. This releases p65 NF-κB (p65/p50), activating the NF-κB pathway and promoting release of inflammatory mediators such as IL-6, IL-1β, and TNF-α, while also activating the downstream NLRP3 (NOD-like receptor pyrin domain-containing protein 3) inflammasome, thereby exerting toxic effects on cellular damage (34).

##### 2.1.4.2 NF-κB pathway affected by copper-based therapeutic strategies

For CuO nanoparticles, a CuO ultrasmall nanozyme-loaded heparin-starPEG hydrogel significantly suppressed NF-κB p65 activity and its downstream signaling by continuously releasing CuO to scavenge ROS and capture MCP-1/CXCL-1 (35, 36). This approach downregulated MCP-1, CXCL-1, and TNF-α levels in wound tissues while upregulating antioxidant proteins such as SOD1 and CAT. By disrupting the ROS-NF-κB-inflammation positive feedback loop, this strategy reduced inflammation and promoted diabetic wound healing in STZ-induced type I diabetic BALB/c mouse dorsal wound models. Additionally, Kanmaru et al. (37) discovered that a Tat peptide conjugated with HPH-Pep-Cu²^+^ also successfully inhibited NF-κB pathway activation by suppressing IκBα phosphorylation. Copper-binding peptides (Cu-bp) and copper-containing fabrics reduce production of inflammatory mediators such as NO and TNF-α by inhibiting nuclear translocation of NF-κB p65 (38, 39).

#### 2.1.5 NF-κB pathway affected by magnesium

Magnesium ions enhance PI3K (phosphatidylinositol 3-kinase)/AKT pathway activation by promoting AKT (protein kinase B) phosphorylation, thereby inhibiting NF-κB pathway activation. This suppresses excessive production of inflammatory mediators, including the cytokines TNF-α and IL-6 and the chemokine MIP-2 (macrophage inflammatory protein 2), thereby attenuating inflammatory responses (40).

Magnesium ions can also directly inhibit TLR4 expression, thereby suppressing lipopolysaccharide-induced NF-κB pathway activation and reducing expression of proinflammatory cytokines IL-6 and IL-8, thus inhibiting the inflammatory response (41). Another study demonstrated that magnesium ions reduce expression of HMGB1, TLR4, NF-κB, and phosphorylated NF-κB in the diaphragm, thereby alleviating the septic inflammatory response in both the diaphragm and serum (42). Additionally, magnesium ions act as calcium antagonists, inhibiting NF-κB pathway activation by reducing intracellular calcium concentrations and thereby decreasing expression of inflammatory cytokines IL-1β, IL-6, and TNF-α (43–45).

In Liang’s study (46), Ti-0.625Mg modulated the TLR-NF-κB axis to induce M1 to M2 macrophage polarization in a Sprague-Dawley rat femoral bone defect model. This upregulated proinflammatory factors (TNF-α, IL-1β) in the early phase and anti-inflammatory factor IL-10 and osteogenic factors BMP-2/6 in the late phase.

#### 2.1.6 NF-κB pathway affected by cobalt

##### 2.1.6.1 NF-κB pathway affected by cobalt ion

Cobalt ions activate TLR4 with the assistance of the co-receptor myeloid differentiation protein 2 (MD2), thereby activating NF-κB and promoting production of proinflammatory factors TNF-α and IL-8, which enhance inflammatory responses (47). In another study, cobalt ions activated TLR4, which in turn activated the NF-κB transcription factor. This promoted secretion of chemokines CCL20 and CXCL10, inducing neutrophil chemotaxis to direct target cells to the site of inflammation, while also enhancing secretion of proinflammatory cytokines IL-6 and IL-8, thereby promoting the inflammatory response (48).

##### 2.1.6.2 NF-κB pathway affected by cobalt-based therapeutic strategies

For cobalt nanoparticles, cobalt in Co-MnNPs (cobalt-manganese nanoparticles) dose-dependently reduced LPS-induced excessive ROS production in mice with bleomycin (BLM)-induced pulmonary fibrosis, thereby inhibiting NF-κB signaling pathway activation and decreasing IL-1β, TNF-α, IL-6, and iNOS expression, ultimately alleviating inflammatory responses. Concurrently, it inhibited fibrosis by suppressing both classical and non-classical TGF-β pathways (49).

#### 2.1.7 NF-κB pathway affected by nickel

##### 2.1.7.1 NF-κB pathway affected by nickel ion

Nickel ions activate the NF-κB pathway by promoting IκBα phosphorylation, thereby increasing expression of IL-1β, IL-6, IL-8, and TNF-α and inducing inflammatory responses in the kidneys and liver (50).

##### 2.1.7.2 NF-κB pathway affected by nickel-based therapeutic strategies

Liu et al. (51) identified that nano nickel oxide (nano NiO) induces NF-κB-activated kinase (NIK), IκB kinase alpha (IKK-α), and NF-κB mRNA while downregulating IκBα. This indicates that nickel oxide nanoparticles activate the NF-κB signaling pathway, increasing levels of proinflammatory cytokines IL-1β and IL-6 while decreasing levels of anti-inflammatory cytokines IL-4 and IL-10. Consequently, they promote inflammation and induce hepatotoxicity.

#### 2.1.8 NF-κB pathway affected by lithium

Monovalent lithium ions upregulate mRNA expression of p50 and IκB-α (NF-κB inhibitory protein alpha) while inhibiting NF-κB translocation from the cytoplasm to the nucleus, thereby suppressing LPS-induced macrophage inflammatory responses and oxidative stress. During LPS-induced inflammation, the NF-κB pathway is activated through dimerization of p65 and p50 subunits, promoting production of inflammatory mediators. Notably, lithium ion-induced upregulation of p50 homodimerization inhibits the transcriptional activity of p50-p65 heterodimers, thereby attenuating inflammatory responses (52).

Lithium-ion treatment reverses spinal cord injury (SCI)-induced overexpression of BDNF-AS (brain-derived neurotrophic factor antisense RNA), thereby promoting BDNF expression in SCI rats. BDNF-AS exerts anti-inflammatory effects by downregulating miR-9-5p expression, inhibiting NF-κB phosphorylation, and suppressing production of proinflammatory factors such as TNF-α, IL-6, and IL-1β (53, 54).

#### 2.1.9 NF-κB pathway affected by Lanthanum

##### 2.1.9.1 NF-κB pathway affected by lanthanum ion

Lanthanum ions are released in gastric acid, forming insoluble lanthanum phosphate particles with phosphate ions that stimulate macrophages (55). Yuan et al. identified that this may promote secretion of inflammatory cytokines IL-1β and IL-8 through the NF-κB pathway, exacerbating inflammatory responses that damage intestinal epithelial cells. In recent tumor immunotherapy research, lanthanum ions activate the NF-κB signaling pathway to block transport of cGAMP-bound STING from the endoplasmic reticulum to the Golgi apparatus and lysosomes, thereby preventing STING degradation and enhancing immune responses (56).

Conversely, lanthanum ions can also exert anti-inflammatory effects. They inhibit PKC/Ca²^+^ pathway activation by suppressing PKCα (protein kinase C) phosphorylation and reducing intracellular Ca²^+^ concentration in lipopolysaccharide (LPS) exposure experiments. Concurrently, they inhibit IκBα degradation, thereby preventing NF-κB/p65 nuclear translocation and suppressing NF-κB pathway activation. This reduces LPS-induced NO and TNF-α production (57). At specific concentrations, lanthanum ions can suppress RANK (receptor activator of NF-κB) expression, thereby affecting the RANKL (receptor activator of NF-κB ligand)/RANK signaling pathway. This inhibits downstream NF-κB expression, suppresses local inflammatory responses, reduces release of inflammatory mediators, ultimately diminishes osteoclast activation, and protects bone tissue (58–60).

##### 2.1.9.2 NF-κB pathway affected by lanthanum-based therapeutic strategies

For lanthanum oxide (61), NF-κB-p65 positivity was observed in the testes of lanthanum oxide-treated mice. Oxidative stress was indicated by decreased SOD activity, accompanied by Bcl-2 downregulation and BAX and Caspase-3 upregulation. In the high-dose group, BAX and Caspase-3 expression showed significant upregulation, suggesting that La_2_O_3_ nanoparticles induce apoptosis and exhibit reproductive toxicity.

#### 2.1.10 NF-κB pathway affected by cerium

##### 2.1.10.1 NF-κB pathway affected by cerium ion

High concentrations of cerium ions activated Nox1 expression in an osteoclastogenesis study, leading to oxidative stress production. This further activated the RANKL signaling pathway, promoting osteoclast differentiation (62). ROS-induced oxidative stress enhances inflammatory cytokine release and RANK pathway activation triggers downstream NF-κB signaling to express TNF-α, IL-1β, and IL-6, these inflammatory mediators are essential for activated osteoclasts, demonstrating that cerium also possesses pro-inflammatory effects. Pang et al. (63) also identified that CdTe QDs activate the NF-κB signaling pathway to upregulate expression of pro-IL-1β, TNF-α, and IL-6.

##### 2.1.10.2 NF-κB pathway affected by cerium-based therapeutic strategies

For cerium oxide nanoparticles, Saif-Elnasr et al. (64) demonstrated that cerium oxide nanoparticles (CONPs) can suppress renal inflammatory injury by reducing levels of NF-κB, TNF-α, and IL-1β in kidney tissue. This may be attributed to downregulation of proinflammatory miR-155 and increased levels of anti-inflammatory miRNAs miR-124 and miR-146a, which negatively regulate expression of TLR6, MyD88, and TNF-α. Furthermore, miR-146a can directly target TLR and its downstream effector molecules IRAK1 and TRAF6, thereby reducing production of inflammatory mediators (65–68). Nano-cerium oxide treatment significantly reduced levels of CRP, ESM-1, and PCT, as well as validated markers TNF-α, IL-6, NF-κB, and LTB4 in a sepsis-induced brain injury study, thereby mitigating encephalitis occurrence (69).

Similarly, hCeO_2_@CuO-based nanoparticles represent a novel anti-inflammatory material (70). Through conversion of Ce³^+^ to Ce^4^^+^ within the nano-cerium oxide core, they scavenge reactive oxygen species (ROS), thereby reducing damage to lysosomal membranes. This inhibits release of cathepsin B (CTSB) from lysosomes, subsequently suppressing NLRP3 activation, reducing inflammasome assembly, and inhibiting caspase-1 activation. Consequently, maturation and secretion of IL-1β and IL-18 are reduced, thereby alleviating inflammatory responses. CTSB induces NF-κB nuclear translocation by promoting IκBα phosphorylation and degradation, thereby upregulating expression of IL-1β, IL-18, and GSDMD-N (71).

#### 2.1.11 NF-κB pathway affected by silver

AgNPs activate the NF-κB pathway. AgNPs promote phosphorylation and degradation of IκBα, the cellular protein that normally sequesters NF-κB in the cytoplasm; loss of IκBα releases NF-κB to translocate to the nucleus and upregulate COX2 (cyclooxygenase-2), thereby promoting inflammatory responses (72–74). Cascione et al. (75, 76) demonstrated that citrate-coated silver nanoparticles (Ag NPs, 5 nm) activate the NF-κB pathway, promoting expression of IL-6 and IL-8 in human leukemia monocyte-derived cells (THP-1) and IL-1α in human cervical cancer cells (HeLa), thereby enhancing inflammatory responses.

Conversely, in Sharma’s study (77), silver nanoparticles synthesized from honeyberry green extract (honeyberry-derived biogenic silver nanoparticles/AgNPs-HB) alleviate cellular inflammatory responses by inhibiting TLR4/MyD88 signaling, which suppresses NF-κB pathway activation.

The apparently contradictory outcomes reported for AgNPs on NF-κB signalling likely reflect differences in nanoparticle surface chemistry, dose, and cellular model rather than a genuine dual mechanism of the silver ion itself. The pro-inflammatory citrate-coated AgNPs (5 nm) used by Cascione et al. carry a small, weakly bound anionic capping ligand that permits rapid Ag^+^ ion release and sustained ROS generation, favouring IκBα degradation and NF-κB-driven cytokine release in transformed monocytic (THP-1) and cervical cancer (HeLa) cell lines at near-cytotoxic concentrations. In contrast, the honeyberry-derived biogenic AgNPs-HB are capped with plant-derived polyphenols and other antioxidant phytochemicals that slow Ag^+^ ion release and directly scavenge ROS, thereby blunting TLR4/MyD88-NF-κB signalling when tested at anti-inflammatory, sub-cytotoxic doses. These observations indicate that the net effect of AgNPs on NF-κB is governed by an interplay of particle size, capping-ligand identity and stability (which dictates Ag^+^ ion-release kinetics), applied concentration, and the redox/inflammatory status of the target cell, rather than by silver acting through two opposing intrinsic mechanisms. Future studies of silver-based immunomodulatory materials should report these parameters systematically to allow direct comparison across AgNP formulations.

#### 2.1.12 NF-κB pathway affected by gold

Yuan et al. (78) demonstrated that peptide-coated gold clusters (Au_25_Sv_9_) inhibit LPS-induced NF-κB activation *in vitro*, thereby suppressing secretion of proinflammatory cytokines such as TNF-α, IL-1β, and IL-6 and attenuating the inflammatory response. Moreover, the peptide-coated gold nanocluster Au_25_Sv_9_ also suppresses overexpression of proinflammatory factors such as TNF-α, IL-6, and iNOS in LPS-stimulated microglia (BV-2) by inhibiting NF-κB pathway activation (79). Furthermore, peptide-coated gold nanoparticles effectively inhibit Toll-like receptor (TLR) signaling in macrophages, thereby suppressing TLR4-mediated activation of the NF-κB signaling pathway. This induces M2 macrophage polarization both *in vitro* and *in vivo*, reduces levels of proinflammatory cytokines such as IL-12/23p40, IL-12p70, and IL-6, and effectively modulates pulmonary inflammation, thereby protecting the lungs from injury and promoting resolution of inflammation (80).

#### 2.1.13 Brief summary of the NF-κB pathway

Metals converge on the NF-κB pathway through markedly different entry points, which explains why some metals predominantly activate the pathway while others predominantly inhibit it, and still others show clearly bidirectional effects. Ferrous ions serve as a typical pro-inflammatory regulator, triggering ROS generation through Fenton-like reactions to facilitate NF-κB pathway activation and inflammatory response escalation. Zinc ions act as a classic anti-inflammatory mediator, suppressing NF-κB signaling by inhibiting IKKβ activity and activating the A20 regulatory protein to alleviate inflammation. Additionally, several metals show bidirectional regulatory characteristics dependent on material forms and experimental conditions: lanthanum ions can either activate or inhibit the NF-κB pathway in different contexts, while silver nanoparticles produce opposing inflammatory regulatory outcomes due to their extrinsic physicochemical properties rather than the intrinsic attributes of silver ions.

### 2.2 MAPK pathway

The mitogen-activated protein kinase (MAPK) signaling pathway is extensively involved in various biological processes, including cell proliferation, differentiation, apoptosis, inflammatory responses, and stress responses, serving as a crucial intracellular signaling system (81). The MAPK pathway primarily comprises three major branches: extracellular signal-regulated kinase (ERK), c-Jun N-terminal kinase (JNK), and p38. Through a cascade of phosphorylation reactions, MAPK transmits extracellular signals to the nucleus, regulating gene expression to influence cellular physiological and pathological states (82).

The MAPK pathway activates transcription factors through stepwise phosphorylation, thereby driving the expression of pro-inflammatory cytokines, chemokines, and inflammatory mediators to initiate and amplify the inflammatory response. Various metal ions can modulate the activity of this pathway. The regulatory roles of various metals in the MAPK pathway are summarized in **Figure 4**.

**Figure 4:**
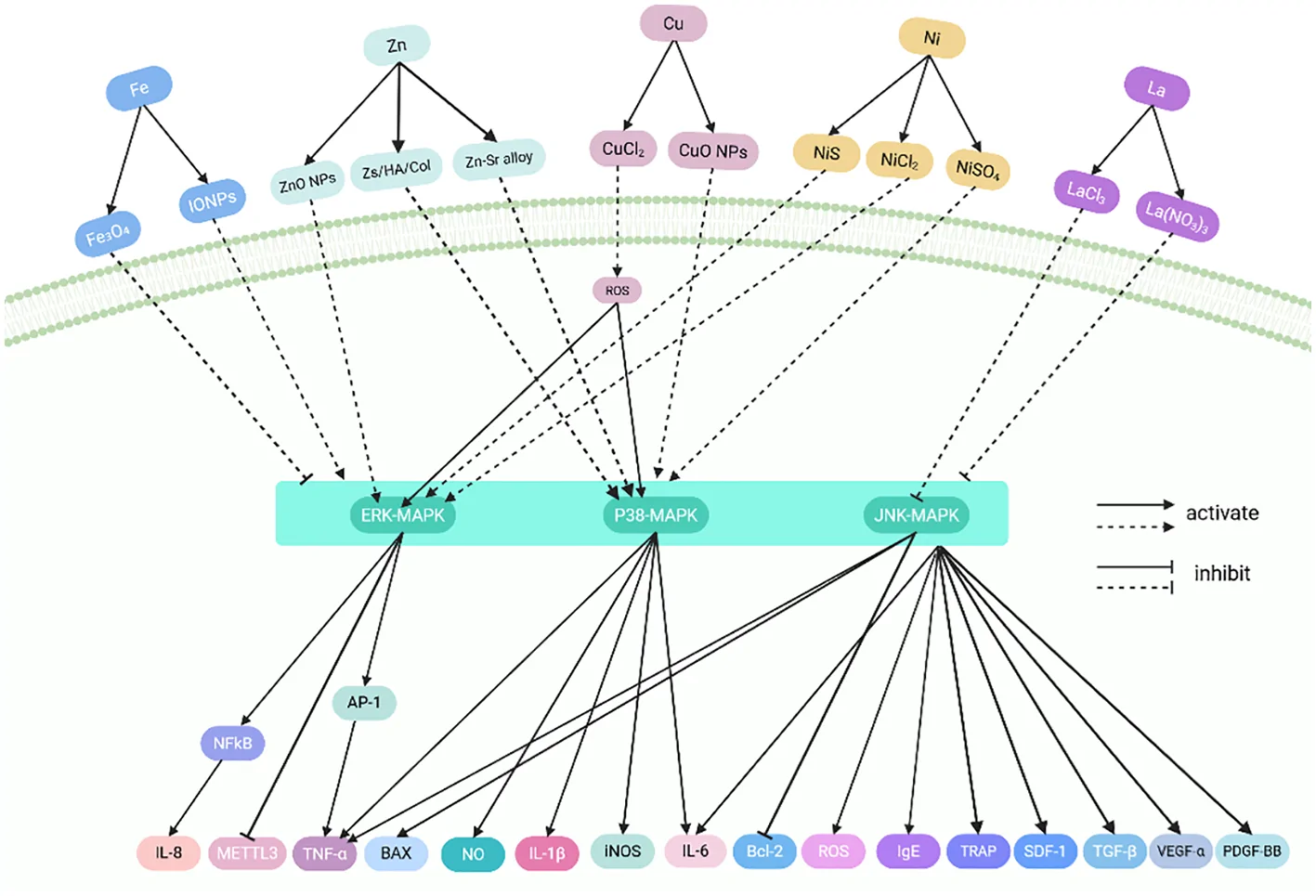
MAPK pathway regulated by Fe, Zn, Cu, Ni and La. MAPK, Mitogen-Activated Protein Kinase; ERK, Extracellular Signal-Regulated Kinase; JNK, c-Jun N-terminal Kinase; p38, p38 Mitogen-Activated Protein Kinase; TNF-α, Tumor Necrosis Factor Alpha; IL-1β, Interleukin-1 Beta; IL-6, Interleukin-6; iNOS, Inducible Nitric Oxide Synthase; MCP-1, Monocyte Chemoattractant Protein-1; ICAM-1, Intercellular Adhesion Molecule 1; MMP, Matrix Metalloproteinase; VEGF, Vascular Endothelial Growth Factor; TGF-β, Transforming Growth Factor Beta; PDGF-BB, Platelet-Derived Growth Factor BB.

#### 2.2.1 MAPK pathway affected by iron

Fe_3_O_4_ in SOD&Fe_3_O_4_@ZIF-8 (SFZ) nanoparticles significantly downregulated p-ERK, p-JNK, and p-p38 phosphorylation levels in the MAPK signaling pathway by modulating oxidative stress and the inflammatory microenvironment (83). In a complete Freund’s adjuvant (CFA)-induced inflammatory pain mouse model, this achieved downregulation of proinflammatory factors TNF-α, IL-6, and IL-1β.Upon entering macrophages, iron oxide nanoparticles (IONPs) activate three branches of the MAPK signaling pathway. IONPs significantly enhance ERK1/2 phosphorylation, thereby inducing the expression of tumor necrosis factor-α (TNF-α); simultaneously, IONPs also activate the JNK signaling pathway, promoting the synthesis and release of interleukin-1β (IL-1β) and TNF-α. Furthermore, IONPs can upregulate p38 MAPK activity, further increasing the expression levels of IL-1β and inducible nitric oxide synthase (iNOS). The three pathways—ERK1/2, JNK, and p38—work synergistically to drive the transformation of macrophages into a pro-inflammatory phenotype (M1), thereby enhancing their bactericidal capacity, phagocytic activity, and migratory ability (84).

#### 2.2.2 MAPK pathway affected by zinc

##### 2.2.2.1 MAPK pathway affected by zinc ion

Zinc ions significantly downregulated phosphorylation levels of JNK and ERK in the MAPK signaling pathway (i.e., suppressed p-JNK/JNK and p-ERK/ERK expression) without affecting the p38 pathway. This intervention downregulated pro-apoptotic factors Bax, caspase-3, and caspase-9 by improving zinc homeostasis and inhibiting oxidative stress, while simultaneously upregulating the anti-apoptotic factor Bcl-2. Consequently, it alleviated bisphenol A (BPA)-induced testicular apoptosis and ferroptosis in a male reproductive toxicity mouse model, ultimately improving reproductive damage in male mice (85).

##### 2.2.2.2 MAPK pathway affected by zinc-based therapeutic strategies

For zinc nanoparticles, zinc gluconate can self-assemble into ZnO nanoparticles within the body, activate the ERK1/2 pathway, and downregulate METTL3 expression (86). Similarly, a zinc silicate/nano-hydroxyapatite/collagen composite scaffold (ZS/HA/Col) releases Zn²^+^, activates p38 MAPK, and upregulates TRAP, SDF-1, TGF-β1, VEGF-α (vascular endothelial growth factor alpha), and PDGF-BB. This recruits BMSCs (bone marrow-derived mesenchymal stem cells) and ECs (endothelial cells), thereby promoting bone regeneration and angiogenesis (87).

Additionally, a biodegradable zinc-strontium alloy (Zn–Sr alloy) releases Zn²^+^, phosphorylates p38 MAPK, and upregulates TNF-α, IL-6, IL-1β, VEGF-α, TGF-β1, and PDGF-BB, thereby promoting the coupling process between osteoclasts and osteoblasts and accelerating bone regeneration and angiogenesis (88).

#### 2.2.3 MAPK pathway affected by copper

##### 2.2.3.1 MAPK pathway affected by copper ion

Copper ions can directly bind to MEK1 to enhance its phosphorylation of ERK1/2, thereby activating ERK1/2 and inducing cancer development (89). Copper chelating agents may enhance efficacy in BRAF V600E-mutated colorectal cancer by inhibiting the MAPK pathway (90). Using the copper ion chelator N-acetylcysteineamide to chelate copper ions across the blood-brain barrier can also alleviate autoimmune encephalomyelitis. By chelating copper ions to reduce reactive oxygen species (ROS) production, it inhibits activation of MAP kinase, JNK, and p38 MAPK, thereby preventing MMP-9-mediated and inflammation-induced neuronal damage (91).

Copper ions in SOD possess the ability to scavenge ROS, yet copper ions themselves can also catalyze the conversion of superoxide anion and hydrogen peroxide into hydroxyl radicals (92). Copper exerts neurotoxic effects similar to those induced by oxidative and inflammatory damage. Under copper chloride stimulation, ROS levels increase, leading to elevated phosphorylation of p38 and ERK1/2. This activates the p38 MAPK and ERK signaling pathways, ultimately resulting in oxidative stress-induced neuronal cell death and apoptosis (93, 94).

##### 2.2.3.2 MAPK pathway affected by copper-based therapeutic strategies

Additionally, CuO nanoparticles exacerbated tissue injury progression by activating JNK, p38 MAPK, and ERK pathways, increasing ROS production, and elevating IgE (immunoglobulin E) levels (95, 96).

#### 2.2.4 MAPK pathway affected by nickel

Nickel ions promote phosphorylation of p38 and JNK MAPK, enhance expression of IL-6 and TNF-α, suppress TGF-β expression, and induce inflammatory responses in human umbilical vein endothelial cells (HUVECs) (97). NiCl_2_/NiS promotes inflammatory responses in human bronchial epithelial cells (Beas-2B) by activating AP-1 (activator protein-1) through ERK activation, thereby inducing TNF-α expression (98). Activated AP-1 can also directly upregulate expression of Gal-1 (galectin-1) and PD-L1 (programmed death-ligand 1), and elevate TNF-α levels by upregulating CCL2 expression (99, 100).

#### 2.2.5 MAPK pathway affected by lanthanum

Lanthanum ions were found to mitigate H_2_O_2_-induced vascular calcification by inhibiting JNK2 and p38 MAPK without affecting ERK1/2. This mechanism reduces secretion of inflammatory factors, thereby inhibiting inflammatory responses and alleviating oxidative stress, which contributes to improving atherosclerosis (101).

Lanthanum ions can also improve dyslipidemia, reduce oxidative stress and vascular endothelial damage, and significantly reduce the size of atherosclerotic plaques. Lanthanum significantly downregulates p38 MAPK phosphorylation levels and reduces the expression of adhesion molecules such as ICAM-1 and MCP-1; simultaneously, it inhibits NF-κB p65 activation and reduces the release of pro-inflammatory factors such as TNF-α, IL-6, and IL-1β. Studies have confirmed that lanthanum exerts its anti-atherosclerotic effects by inhibiting the p38 MAPK pathway, downregulating inflammatory and adhesion molecules, and suppressing vascular inflammatory responses (102). Lanthanum ions exert their effects by activating the ERK and JNK MAPK pathways, thereby inhibiting the osteogenic differentiation and calcification of vascular smooth muscle cells, and providing a new target for interventions against vascular calcification (103).

#### 2.2.6 Brief summary of the MAPK pathway

Metals regulate the MAPK cascade through a mixture of consistently one-directional and context-dependent bidirectional actions, so that some elements behave as straightforward activators while others switch direction depending on which branch, cell type or formulation is involved. Copper and nickel act as consistent MAPK activators: Cu²^+^ both binds MEK1 directly to enhance ERK1/2 phosphorylation and, via ROS generation, activates p38 and ERK1/2 in neuronal and pulmonary tissue (an effect reversed by copper chelation), while Ni²^+^ promotes p38/JNK phosphorylation directly and drives ERK-AP-1 signalling in epithelial cells, in both cases raising pro-inflammatory cytokine output. Iron, zinc and lanthanum instead show branch- and context-dependent bidirectionality. Iron oxide nanoparticles (IONPs) activate all three MAPK branches (ERK, JNK, p38) to drive M1 macrophage polarization, whereas an antioxidant SOD-Fe_3_O_4_@ZIF-8 nanozyme lowers oxidative stress and thereby suppresses the same phosphorylation events. Zinc ions dampen JNK/ERK phosphorylation in a reproductive-toxicity model, yet zinc-releasing nanomaterials and bone scaffolds instead activate ERK1/2 or p38 to support osteogenic and angiogenic signalling. Lanthanum predominantly inhibits JNK2 and p38 (sparing ERK1/2) in vascular-calcification and atherosclerosis models, but in a separate calcification study it instead activates ERK/JNK to suppress osteogenic differentiation of vascular smooth muscle cells. This pattern indicates that the net direction of MAPK modulation depends less on the identity of the metal than on which branch is engaged, the cell type involved, and whether the metal is delivered as a free ion, a redox-active nanoparticle, or an antioxidant-functionalised formulation.

### 2.3 AKT pathway

The PI3K/Akt signaling pathway serves as a key intracellular signaling network extensively involved in various biological processes, including cell proliferation, survival, metabolism, and migration. Activated by receptor tyrosine kinases (RTKs) or G protein-coupled receptors, PI3K catalyzes the conversion of PIP2 into PIP3. As a second messenger, PIP3 recruits AKT to the cell membrane, promoting its phosphorylation at Thr308 by PDK1 (phosphoinositide-dependent kinase 1) and at Ser473 by mTORC2 (mammalian target of rapamycin complex 2) (104). AKT activation not only regulates cell cycle and metabolism but also plays a crucial role in inflammation, immune modulation, and tumorigenesis (105). Different AKT subtypes (AKT1, AKT2, AKT3) exhibit distinct tissue distribution and functional characteristics, conferring high regulatory specificity to this signaling pathway. Given the central role of the PI3K/Akt pathway in multiple diseases, therapeutic strategies targeting this pathway represent a key direction for treating immune-related disorders. The regulatory roles of various metals in the AKT pathway are summarized in **Figure 5**.

**Figure 5:**
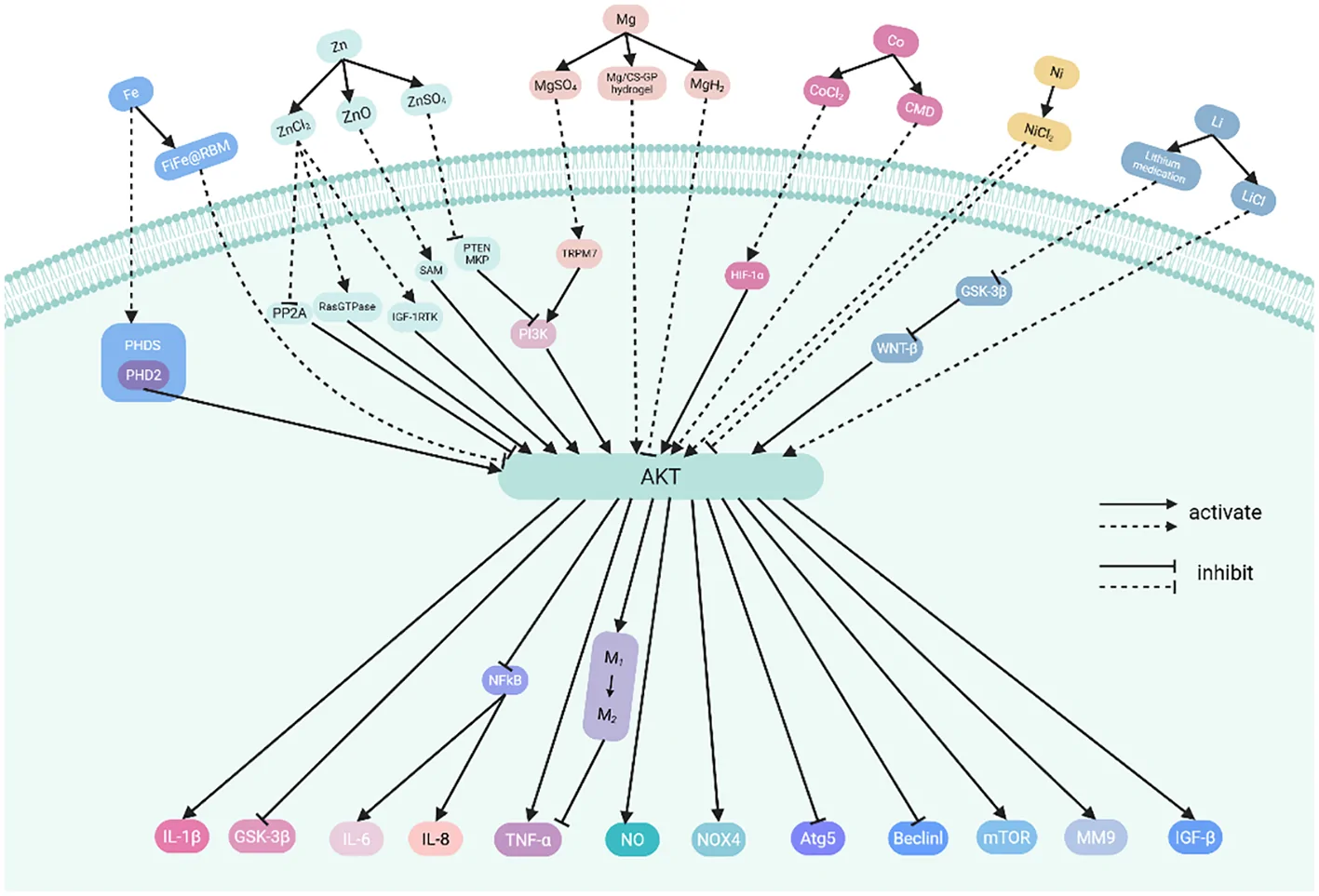
AKT pathway regulated by Fe, Zn, Mg, Co, Ni, and Li. PI3K, Phosphoinositide 3-Kinase; AKT, Protein Kinase B; mTOR, Mammalian Target of Rapamycin; mTORC2, Mammalian Target of Rapamycin Complex 2; PTEN, Phosphatase and Tensin Homolog; PIP2, Phosphatidylinositol 4, 5-bisphosphate; PIP3, Phosphatidylinositol 3, 4,5-trisphosphate; PDK1, Phosphoinositide-Dependent Kinase-1; GSK-3β, Glycogen Synthase Kinase-3 Beta; FOXO, Forkhead Box O Transcription Factors; Bad, BCL2-Associated Agonist of Cell Death; RTK, Receptor Tyrosine Kinase; ROS, Reactive Oxygen Species.

#### 2.3.1 AKT pathway affected by iron

##### 2.3.1.1 AKT pathway affected by iron ion

Iron (Fe²^+^) is an essential cofactor for PHDs (prolyl hydroxylase domain proteins), a class of critical oxygen-sensing enzymes. Simultaneously, PHD2 hydroxylates Akt1/Akt2, promoting Akt binding to pVHL (von Hippel-Lindau protein). pVHL then recruits protein phosphatase PP2A, leading to dephosphorylation of Akt at Thr308 and subsequent Akt inactivation (106). In hypoxic or pVHL-deficient cancer cells, PHD2 is inactivated, leading to excessive Akt activation and promoting thrombosis and inflammation (107, 108).

##### 2.3.1.2 AKT pathway affected by iron-based therapeutic strategies

Therefore Cheng et al. (109) reported a FASN inhibitor and iron oxide nanoparticles encapsulated in red blood cell membranes (FiFe@RBM) inhibited the AKT-mTOR pathway by releasing Fe²^+^/Fe³^+^ ions, inducing reactive oxygen species (ROS) and mitochondrial dysfunction, ultimately leading to a synergistic effect of apoptosis and ferroptosis.

#### 2.3.2 AKT pathway affected by zinc

##### 2.3.2.1 AKT pathway affected by zinc ion

Zinc ions can reduce Akt dephosphorylation by inhibiting PP2A (serine/threonine protein phosphatase), thereby maintaining Akt in its activated state. At low molar concentrations, zinc ions activate the Akt signaling pathway via Ras GTPase (110). Zinc sulfate degrades PTEN (phosphatase and tensin homolog) via the proteasome pathway, thereby activating the PI3K/Akt pathway (111). Additionally, under conditions not involving PTEN, zinc ions can activate the Akt signaling pathway by activating IGF-1R TK (insulin-like growth factor 1 receptor tyrosine kinase), thereby transmitting cardioprotective signals (112).

##### 2.3.2.2 AKT pathway affected by zinc-based therapeutic strategies

For ZnO nanoparticles, zinc oxide (ZnO) increases intracellular SAM levels by activating the ZNG1-METAP1 complex. This subsequently promotes AKT methylation at residues R15 and R391 via PRMT5-mediated methylation, facilitating AKT translocation from the cytoplasm to the cell membrane and its subsequent activation. In DSS-induced colitis mouse models, this process significantly upregulates expression of anti-inflammatory factors IL-4 and TGF-β while downregulating proinflammatory factors TNF-α, IL-6, IL-1β, IL-12, IFN-γ, TLR2, and TLR4, thereby improving intestinal barrier function and alleviating intestinal inflammation (113).

#### 2.3.3 AKT pathway affected by magnesium

##### 2.3.3.1 AKT pathway affected by magnesium ion

Magnesium ions can activate the kinase activity of TRPM7 (transient receptor potential melastatin 7). By activating TRPM7 kinase activity, magnesium promotes PI3K phosphorylation, thereby activating the AKT pathway. This significantly increases mRNA expression of chemotactic factors MMP2, MMP9, and VEGF, promoting cell growth and survival (114, 115).

##### 2.3.3.2 AKT pathway affected by magnesium-based therapeutic strategies

In Shi’s study (116), magnesium hydride suppressed activation of LPS-induced AKT/mTOR and NF-κB/NLRP3 pathways, inhibited oxidative stress *in vivo*, and reduced production of the proinflammatory cytokine IL-1β, thereby attenuating the inflammatory response. Additionally, Jiang et al. (117) found that magnesium particles combined with β-glycerophosphate (β-GP) and chitosan (CS) form an injectable porous hydrogel system. The Mg/CS–GP hydrogel (magnesium particles/chitosan–β-glycerophosphate hydrogel) delivers magnesium ions that activate the PI3K/Akt signaling pathway, thereby inhibiting production of TNF-α and IL-1 and suppressing the inflammatory response.

#### 2.3.4 AKT pathway affected by cobalt

Cobalt ions stabilize hypoxia-inducible factor 1α (HIF-1α). As a key hypoxia-responsive transcription factor, stabilized HIF-1α accumulates within cells, thereby mimicking hypoxic conditions. Hypoxia promotes AKT phosphorylation, activating the PI3K/AKT signaling pathway and consequently enhancing cell proliferation (118, 119). Ton et al. (120) identified that inhalation of cobalt metal dust (CMD) activates the PI3K/AKT signaling pathway, promotes NADPH oxidase 4 (NOX4) gene expression, exacerbates oxidative stress levels, and induces carcinogenesis in mouse lung cells.

#### 2.3.5 AKT pathway affected by nickel

Exposure to nickel ions in the kidney inhibits phosphorylation of PI3K and AKT, thereby suppressing the PI3K/AKT pathway and subsequently reducing mTOR expression. mTOR serves as a key negative regulator of autophagy. Treatment with nickel ions promotes autophagy by inhibiting the PI3K/AKT/mTOR pathway, leading to increased mRNA and protein levels of autophagy-related proteins such as Beclin1 and Atg5 (autophagy-related gene 5), along with an increase in autophagolysosome numbers. This ultimately induces autophagy, resulting in renal injury (121).

Additionally, nickel ions exacerbated bleomycin-induced pulmonary fibrosis in mouse models. In pulmonary fibroblasts, nickel ions induced AKT phosphorylation, significantly promoting increased levels of IL-1β, TGF-β, and TNF-α, while simultaneously stimulating fibroblast proliferation and collagen/MMP9 production, thereby exacerbating bleomycin-induced pulmonary inflammation and fibrosis (122).

#### 2.3.6 AKT pathway affected by lithium

Lithium ions can alleviate glucocorticoid-induced chondrocyte autophagy by activating the PI3K/AKT/mTOR signaling pathway through promoting phosphorylation of AKT and mTOR (123). Additionally, lithium ions promote WNT/β-catenin expression by inhibiting GSK-3β synthesis. GSK-3β serves as the primary inhibitor of the WNT/β-catenin pathway. WNT/β-catenin reduces NF-κB signaling activity by promoting activation of the PI3K/AKT pathway, subsequently reducing expression of IL-6, IL-8, and TNF-α, thereby suppressing inflammatory responses (124).

#### 2.3.7 Brief summary of the AKT pathway

Across the AKT pathway, metals again divide into consistent activators and metals whose net effect depends on tissue or formulation context. Zinc, cobalt and lithium act as consistent AKT activators through distinct upstream mechanisms: Zn²^+^ inhibits the phosphatases PP2A and PTEN and engages Ras- and IGF-1R-dependent inputs to sustain Akt phosphorylation, and ZnO nanoparticles separately promote AKT methylation to strengthen the intestinal barrier in colitis; Co²^+^ stabilises HIF-1α, pharmacologically mimicking hypoxia to drive Akt phosphorylation in both physiological proliferation and cobalt-dust-induced carcinogenesis; and Li^+^ activates AKT/mTOR directly and, separately, inhibits GSK-3β to promote WNT/β-catenin-dependent AKT activation, thereby lowering NF-κB-driven cytokine output. Iron, magnesium and nickel are instead bidirectional. Fe²^+^ normally serves as a PHD2 cofactor that restrains Akt via the pVHL-PP2A axis, so hypoxia or PHD2 loss causes excessive Akt activation and thrombo-inflammatory injury, whereas iron-oxide nanoparticles instead inhibit the AKT-mTOR axis to induce ferroptosis/apoptosis. Mg²^+^ activates AKT through TRPM7-PI3K signalling and through Mg-releasing hydrogels that dampen TNF-α/IL-1, but magnesium hydride instead suppresses AKT/mTOR (alongside NF-κB/NLRP3) to lower IL-1β. Ni²^+^ inhibits PI3K/AKT/mTOR in the kidney, triggering autophagy-associated injury, yet activates AKT in pulmonary fibroblasts to promote fibrosis. This suggests that a metal’s net effect on AKT depends on which upstream regulator (PTEN, TRPM7, PHD2/HIF, or mTOR itself) predominates in the tissue or nanoparticle system under study, rather than on a fixed intrinsic action of the ion.

### 2.4 STAT pathway

The STAT (signal transducer and activator of transcription) signaling pathway plays a central role in regulating various physiological and pathological processes, including immunity, inflammation, and tumorigenesis. This pathway is typically activated by cytokines such as IL-6 and IFN-γ, relying on JAK (Janus kinase) to mediate phosphorylation, dimerization, and nuclear translocation of STAT proteins, thereby regulating transcription of target genes (125). In recent years, metallic nanomaterials such as iron, zinc, copper, nickel and lithium have been demonstrated to influence the STAT signaling pathway through multiple mechanisms. The regulatory roles of various metals in the STAT pathway are summarized in **Figures 6**, **7**.

**Figure 6:**
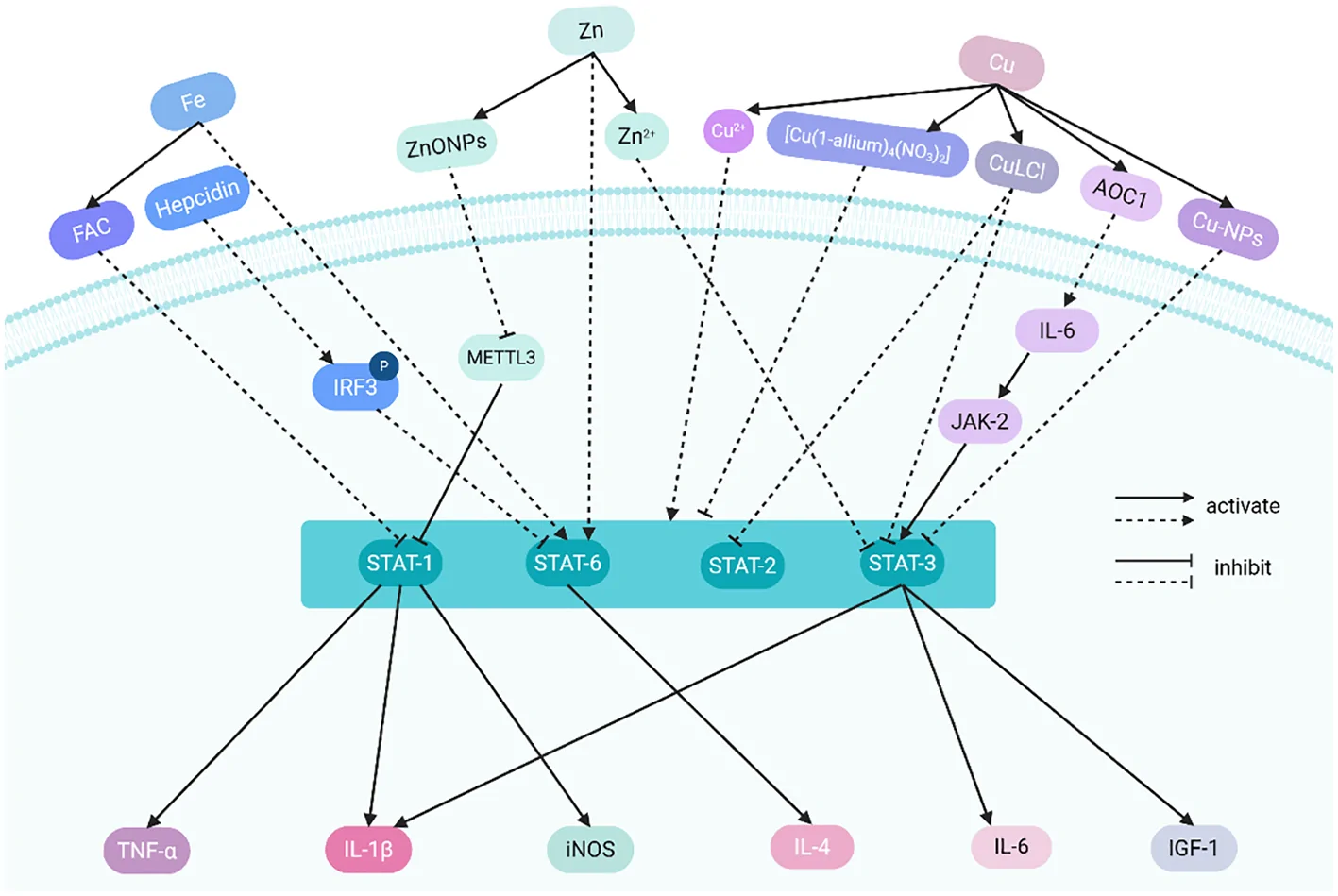
STAT pathway regulated by Fe, Zn and Cu. STAT, Signal Transducer and Activator of Transcription; JAK, Janus Kinase; IL-6, Interleukin-6; IFN-γ, Interferon Gamma; IL-6R, Interleukin-6 Receptor; TNF-α, Tumor Necrosis Factor Alpha; iNOS, Inducible Nitric Oxide Synthase.

**Figure 7:**
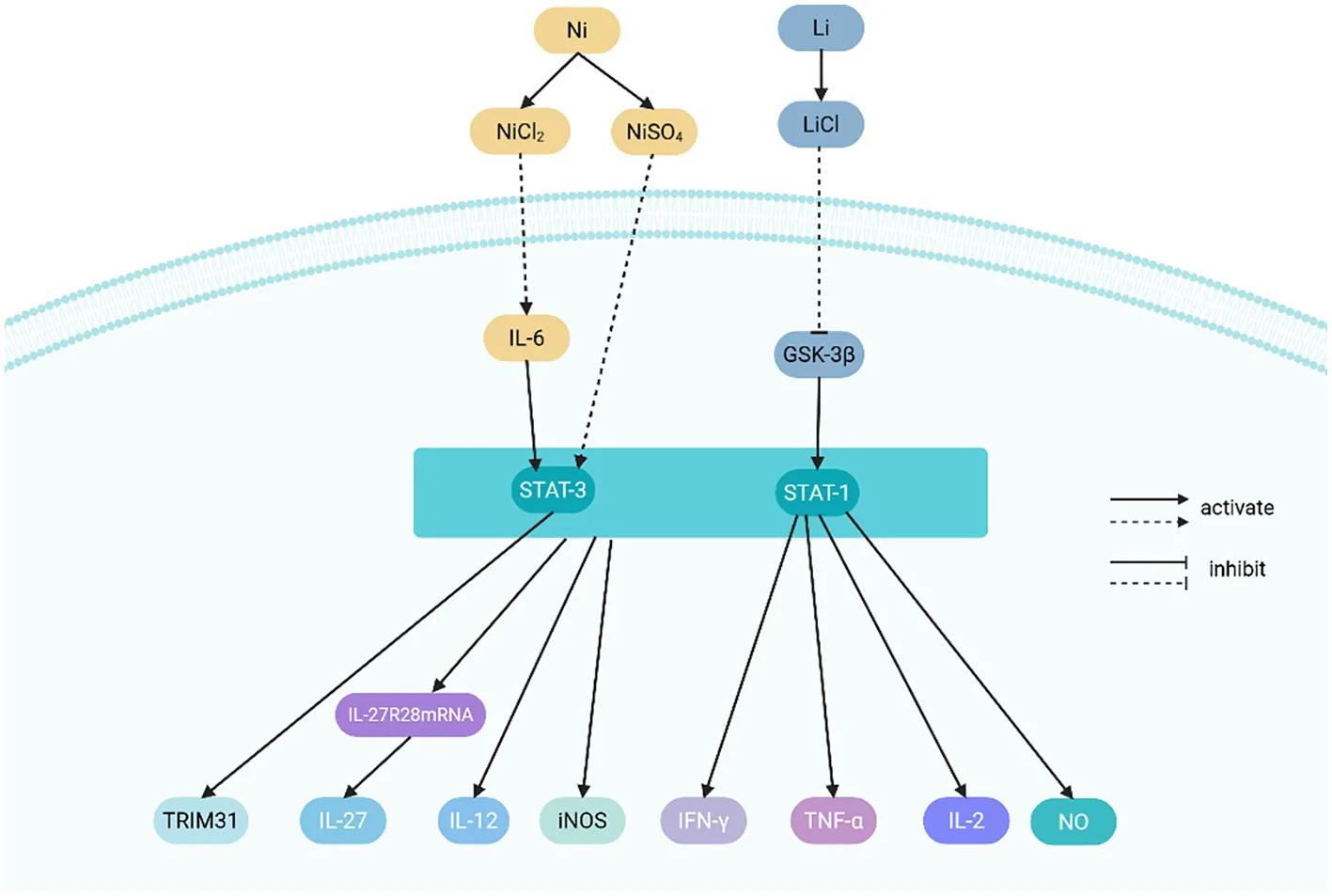
STAT pathway regulated by Ni and Li. STAT, Signal Transducer and Activator of Transcription; JAK, Janus Kinase; IL-6, Interleukin-6; IL-6R, Interleukin-6 Receptor; IFN-γ, Interferon Gamma; TNF-α, Tumor Necrosis Factor Alpha; IL-2, Interleukin-2.

#### 2.4.1 STAT pathway affected by iron

Free iron ions do not directly activate STAT themselves but amplify IL-6R/JAK1/STAT3 signaling through IL-6, leading to increased STAT3 phosphorylation (126). This subsequently upregulates HIF-1α and DMT1, ultimately elevating inflammatory mediators such as IL-6 and TNF-α in a BAPN Ang II-induced acute aortic dissection C57BL/6 mouse model. Berton et al. identified that binding of IL-6 to its receptor activates JAK2, enabling it to phosphorylate the downstream target STAT. Phosphorylated STAT then dimerizes and translocates to the nucleus, where it activates transcription of the ferritin gene, promoting ferritin expression (127, 128).

Blocking IL-6R or chelating iron can both downregulate p-STAT3 and downstream inflammatory pathways (129). Liu et al. (130) found that ferric ammonium citrate (FAC) suppresses inflammatory responses by inhibiting expression of IL-1β, iNOS, and TNF-α through blocking STAT signaling.

#### 2.4.2 STAT pathway affected by zinc

Zinc ions can restore zinc homeostasis in the prostate, significantly inhibit the activation of the JAK1/STAT3 pathway, downregulate the expression of IL-1β, IL-6, and IGF-1, and alleviate inflammatory infiltration and excessive cell proliferation. At the same time, zinc ions promote apoptosis by upregulating caspase-3/8 and Bax and downregulating Bcl-2. In high-fat diet-induced obese rats with prostate hyperplasia and inflammatory damage, obesity leads to a significant increase in pro-inflammatory factors such as IL-1β, IL-6, and IGF-1 in prostate tissue, activates the JAK1/STAT3 pathway, and triggers inflammation and epithelial hyperplasia (131).

The release of Zn²^+^ ions into the cytoplasm inhibits ZIP6 protein expression and activates the JAK1/STAT1/MMP-10 signaling pathway. Under hypoxic microenvironments, increased mitochondrial permeability in bone marrow mesenchymal stem cells (BMSCs) leads to the release of intracellular zinc ions into the cytoplasm. Zinc supplementation promotes MMP-10 expression and angiogenesis in the early stage and facilitates osteogenic differentiation of BMSCs in the later stage, thereby stably improving the repair efficacy of bone defects (132).

Additionally, Gammoh et al. (133) reported that free zinc promotes STAT6 phosphorylation, thereby enhancing IL-4 production and exerting anti-inflammatory effects, inhibiting the production of pro-inflammatory cytokines TNF-α and IL-6. Zinc also enhances TGF-β1-dependent regulatory T cell (Treg) induction and indirectly suppresses Th1-type immune responses, thereby reducing the release of IFN-γ.

#### 2.4.3 STAT pathway affected by copper

In hepatocellular carcinoma (HCC), copper-dependent amine oxidase 1 (AOC1) is upregulated, where it enhances IL-6 expression, activates JAK2, further phosphorylates STAT3, and promotes cell proliferation and migration (134). In the A549 lung cancer cell line, treatment with the 1-allyl imidazole copper complex [Cu(1-allim)_4_(NO_3_)_2_] downregulated interferon receptor subunit 1 (IFNAR1), the interleukin-6 signal transducer gene (IL6ST), and platelet-derived growth factor receptor alpha (PDGFRA), but upregulated SOCS1, SOCS2, SOCS5, and JAK-STAT-related negative regulators. This inhibited the STAT pathway, leading to cell cycle arrest and apoptosis (135). In the AR-230 leukemia model, treatment with the copper complex [CuLCl] of 1H-benzimidazol-2-ylhydrazone significantly inhibited STAT2/3 phosphorylation and suppressed STAT pathway activation compared to the reference drug imatinib (136).

#### 2.4.4 STAT pathway affected by nickel

Exposure to nickel ions induces IL-6 expression, which indirectly leads to STAT3 phosphorylation. Phosphorylated STAT3 translocates to the nucleus, where it mediates transcriptional regulation of TRIM31 (tripartite motif-containing protein 31), thereby increasing TRIM31 expression and promoting invasion and metastasis in cancer cells (137).

Additionally, nickel ions promote activation of the Jak-STAT pathway in human monocyte-derived dendritic cells (MoDCs), leading to increased expression of IL-27 p28 mRNA and enhanced IL-27 production. This regulates IL-22 production by human CD4^+^ T cells, promotes inflammatory responses, and results in allergic contact dermatitis (ACD) (138).

#### 2.4.5 STAT pathway affected by lithium

The most widely recognized mechanism of lithium ions involves inhibiting glycogen synthase kinase-3β (GSK-3β), a kinase widely expressed in tissues and associated with inflammation. Lithium ions inhibit STAT1 phosphorylation by suppressing GSK-3β, thereby suppressing Th1 cell production and secretion of IFN-γ, TNF-α, and IL-2, ultimately alleviating inflammatory responses (139, 140).

#### 2.4.6 Brief summary of the STAT pathway

Metal regulation of STAT signalling shows a similar split: nickel and lithium act in a single, consistent direction, while iron, zinc and copper instead switch direction with cellular or disease context. Nickel consistently activates STAT signalling, inducing IL-6-driven STAT3 phosphorylation that promotes TRIM31-mediated cancer invasion and separately driving JAK-STAT activation in dendritic cells to raise IL-27/IL-22 output in allergic contact dermatitis. Lithium consistently inhibits STAT signalling, suppressing GSK-3β to block STAT1 phosphorylation and downstream Th1 cytokine (IFN-γ/TNF-α/IL-2) secretion. Iron, zinc and copper are instead bidirectional. Iron amplifies IL-6R/JAK1/STAT3 signalling to raise IL-6/TNF-α, while iron chelation or ferric ammonium citrate instead suppresses STAT-driven IL-1β/iNOS/TNF-α. Zinc inhibits JAK1/STAT3 to lower IL-1β/IL-6/IGF-1 in a model of prostatic inflammation, yet separately activates JAK1/STAT1/MMP-10 signalling to support angiogenesis and osteogenic repair, and promotes STAT6 phosphorylation to drive an anti-inflammatory IL-4 response. Copper-dependent AOC1 activates JAK2/STAT3 to promote hepatocellular carcinoma proliferation, whereas copper complexes elsewhere upregulate SOCS negative regulators to inhibit STAT2/3 phosphorylation and induce apoptosis. As with NF-κB and AKT, these findings indicate that the same metal can occupy opposite positions on the STAT axis depending on which STAT isoform is engaged and whether the surrounding signalling context favours cytokine amplification or negative-feedback induction.

### 2.5 Oxidative stress

Reactive oxygen species (ROS), as by-products of cellular metabolism, are both key regulators of cellular signalling molecules and important mediators of oxidative stress. By modulating various signalling pathways, including MAPKs, PI3K/AKT and NF-κB, ROS are involved in cellular processes such as cell proliferation, apoptosis, inflammation and metabolic regulation. When ROS accumulate excessively, intracellular biomolecules suffer oxidative damage, thereby triggering inflammatory responses and tissue damage (141). Conversely, moderate levels of ROS can enhance cellular antioxidant defenses by activating the Nrf2 (nuclear factor erythroid 2-related factor 2) pathway, thereby mitigating oxidative damage. Furthermore, ROS-mediated signaling involves redox modifications of metabolic enzymes, regulating processes such as glycolysis and the tricarboxylic acid cycle, thereby influencing cellular energy metabolism and immune function (142). Numerous metal ions can significantly influence ROS levels. The regulatory roles of various metals in oxidative stress are summarized in **Figures 8**, **9**.

**Figure 8:**
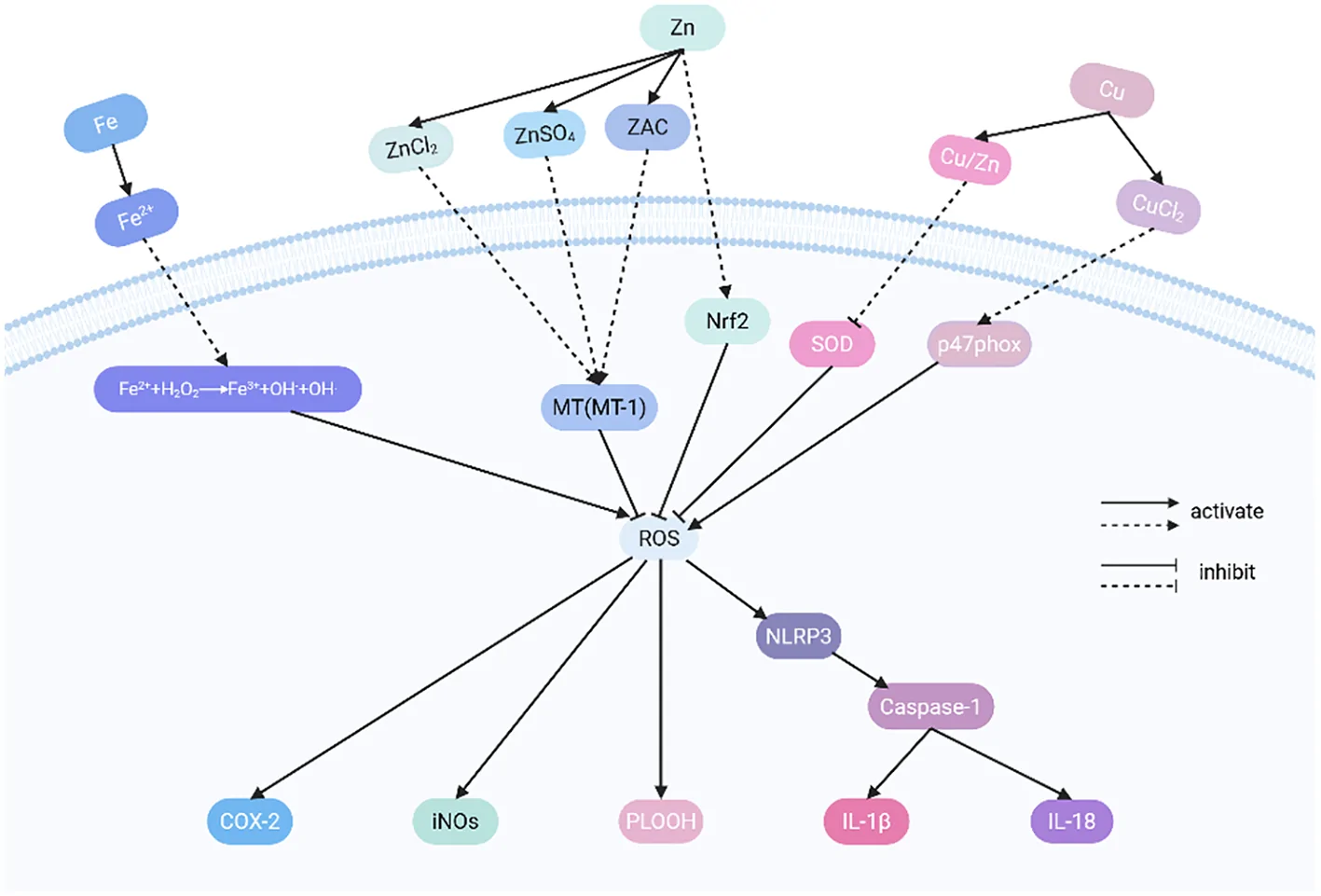
ROS pathway regulated by Fe, Zn and Cu. SOD, Superoxide Dismutase; GPX4, Glutathione Peroxidase 4; Nrf2, Nuclear Factor Erythroid 2-Related Factor 2; HO-1, Heme Oxygenase-1; NADPH, Nicotinamide Adenine Dinucleotide Phosphate; NOX, NADPH Oxidase; TCA, Tricarboxylic Acid Cycle; DLAT, Dihydrolipoyl Transacetylase; Fe-S, Iron-Sulfur Cluster; ROS, Reactive Oxygen Species; GSH, Glutathione (reduced); GSSG, Glutathione Disulfide (oxidized); MDA, Malondialdehyde.

**Figure 9:**
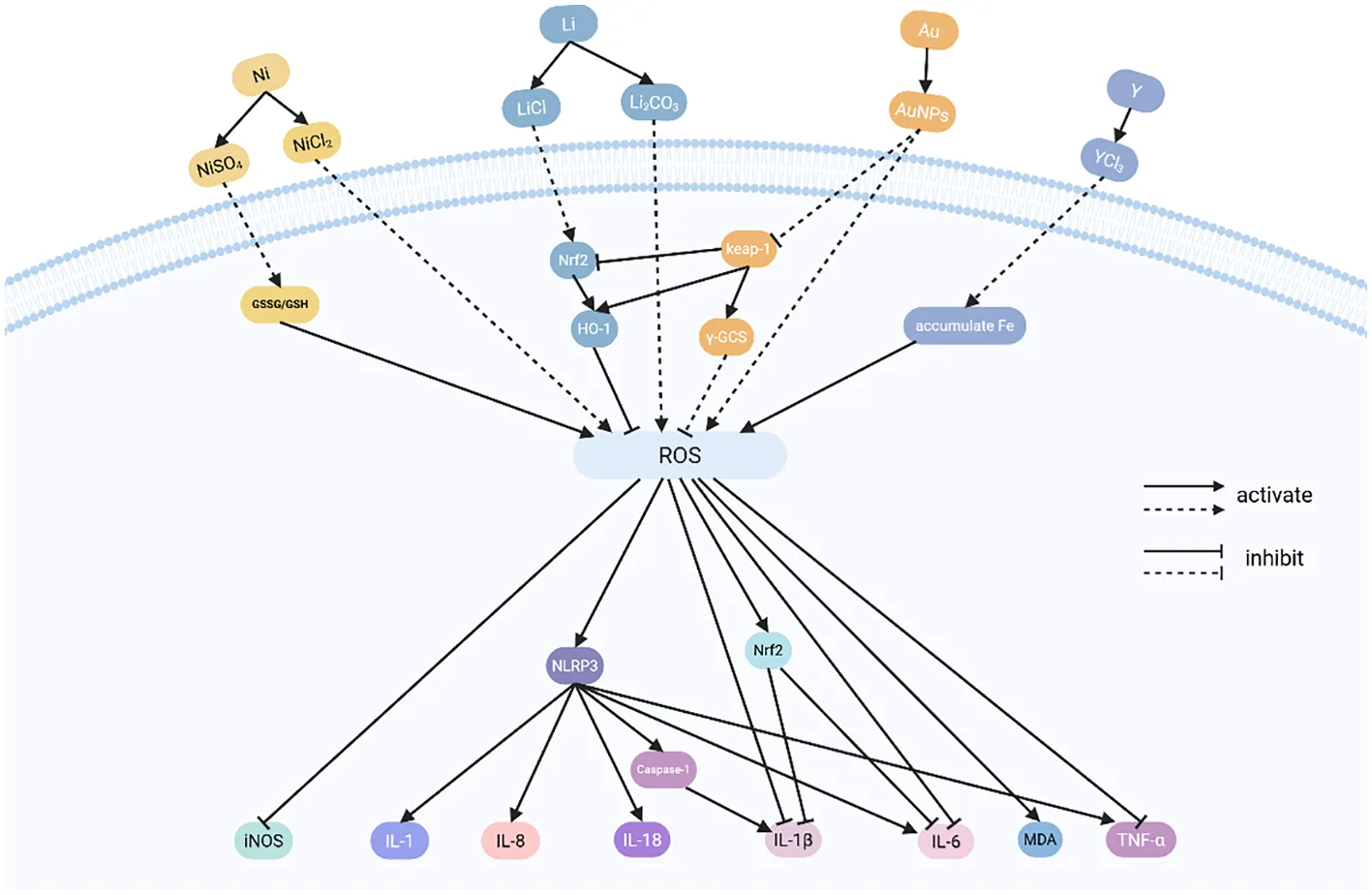
ROS pathway regulated by Ni, Li, Au and Y. Nrf2, Nuclear Factor Erythroid 2-Related Factor 2; Keap1, Kelch-like ECH-Associated Protein 1; HO-1, Heme Oxygenase-1; SOD, Superoxide Dismutase; NLRP3, NOD-like Receptor Pyrin Domain-Containing Protein 3; ROS, Reactive Oxygen Species; GSH, Glutathione; MDA, Malondialdehyde; IL-1β, Interleukin-1 Beta; TNF-α, Tumor Necrosis Factor Alpha.

#### 2.5.1 Oxidative stress affected by iron

##### 2.5.1.1 Oxidative stress affected by iron ion

Ferritin is the primary regulator of iron metabolism, and the regulation of ferritin expression by iron levels constitutes a feedback mechanism for iron homeostasis (143). Excess iron stimulates ferritin expression, reducing cellular iron uptake; conversely, iron deficiency suppresses ferritin, thereby increasing cellular iron uptake and elevating intracellular iron concentrations. Ferritin regulates plasma iron concentration by controlling the concentration of transferrin on iron-exporting cells (144). It induces downregulation of transferrin, leading to elevated iron levels within cells and reduced plasma iron levels, which can prevent iron overload disease (145).

Under the influence of ferrous ions, catalysis of unsaturated fatty acids highly expressed on cell membranes induces lipid peroxidation, thereby triggering cell death (146). Ferroptosis is a novel iron-dependent form of programmed cell death. Imbalances between oxidant production and antioxidant levels due to redox system abnormalities lead to lipid peroxide accumulation and trigger ferroptosis. The Xc^-^-GPX4, FSP1 (ferroptosis suppressor protein 1)-CoQ10 (coenzyme Q10) (147), GCH1 (GTP cyclohydrolase 1)-BH4 (tetrahydrobiopterin) (148), and DHODH (dihydroorotate dehydrogenase)-CoQ10 pathways (149) are the four established antioxidant pathways associated with ferroptosis (15). Ferrous ions enter cells and undergo the Fenton reaction, continuously generating hydroxyl radicals and oxygen radicals. This leads to accumulation of lipid peroxidation products such as phospholipid hydroperoxide (PLOOH), ultimately causing ferroptosis (150).

##### 2.5.1.2 Oxidative stress affected by iron-based therapeutic strategies

Therefore, SP-PFe(SPEEK/PDA-PS/PLGA@Fe) implants can generate sulfate radicals (·SO_4_^-^) and hydroxyl radicals (·OH) without relying on external energy or endogenous oxygen (O_2_) or hydrogen peroxide (H_2_O_2_) (151). These radicals belong to reactive oxygen species (ROS) and effectively eliminate bacteria. Additionally, Fe²^+^ can penetrate bacterial cells, inducing ferroptosis-like phenomena through lipid peroxidation, thereby further enhancing antimicrobial efficacy.

#### 2.5.2 Oxidative stress affected by copper

Excess copper ions bind to dihydrolipoyl transacetylase (DLAT) in the TCA (tricarboxylic acid) cycle, causing protein aggregation and inactivation. This leads to destruction of Fe-S proteins, disruption of the oxidative respiratory chain, and ultimately cell death. Cuproptosis, a newly discovered form of cell death distinct from apoptosis, is mitochondria-dependent and involves the copper ion transporter elesclomol (ES), which transports copper ions into cells, increasing intracellular copper concentration (152–154). Unlike ferroptosis, which involves Fe²^+^ accumulation and ROS production, cuproptosis is characterized by DLAT aggregation and direct disruption of mitochondrial metabolism (155, 156).

Copper chloride promotes ROS generation by activating p47phox, a key component of the NADPH oxidase (NOX) system. ROS then activate the NLRP3 inflammasome, leading to caspase-1 activation and enhanced secretion of IL-1β and IL-18. This demonstrates that copper ions exert pro-inflammatory effects by promoting macrophage polarization toward the M1 phenotype. Studies using TNBS-induced colitis demonstrate that increased expression of the cellular copper chaperone antioxidant-1 (Atox1) promotes copper transport (157). In addition to Atox1, copper can promote macrophage polarization toward the proinflammatory M1 phenotype through signaling via copper transporter 1 (CTR1) and ATP7A (copper-transporting ATPase alpha) (158).

Cu/Zn-SOD, as the earliest discovered SOD family protein, shoulders the crucial task of eliminating excess ROS to maintain intracellular homeostasis, ensuring normal cellular function and survival while providing antioxidant defense (159–161). Copper is an essential cofactor in Cu/Zn-SOD, whose catalytic activity depends on the catalytic role of copper ions. Copper ions participate in electron transfer at the enzyme’s active site, converting superoxide anion—generated by mitochondria and other sources—into oxygen and hydrogen peroxide through inner and outer sphere electron transfers. Cu/Zn-SOD is distributed in the cytoplasm, lysosomes, and the intermembrane space of mitochondria (162). Currently, leveraging the antioxidant properties of Cu/Zn-SOD, bioproduction of exogenous Cu/Zn-SOD, and modification of Cu/Zn-SOD analogues are demonstrating significant potential in treating oxidative stress-related diseases (163). Macrophage polarization is associated with ROS levels, and reducing ROS production can inhibit M1 polarization of macrophages, which plays a significant role in treating spinal cord nerve injuries (164, 165). Conversely, leveraging the proinflammatory effects of macrophage polarization and activation can be exploited for cancer therapy (166).

#### 2.5.3 Oxidative stress affected by zinc

Zinc can stimulate expression of Nrf2, a key regulator of the antioxidant system that suppresses reactive oxygen species production and inhibits inflammatory responses (167). Additionally, Zinc ions significantly upregulate expression of MT1 family genes (168, 169). The MT-1 and MT-2 subtypes can suppress ROS, thereby indirectly inhibiting synthesis of proinflammatory enzymes cyclooxygenase-2 (COX-2) and inducible nitric oxide synthase (iNOS) (170), ultimately attenuating the inflammatory response.

Zn²^+^ can bind to free sulfhydryl groups in proteins, preventing their oxidation and thereby protecting protein structure and function from oxidative damage. Zn²^+^ can also react with cysteine residues in proteins to form zinc thiolates. This structure can release free Zn²^+^ upon encountering oxidants, generating a zinc signal that triggers the cell’s antioxidant response (171).

#### 2.5.4 Oxidative stress affected by nickel

Nickel ions increase the intracellular ratio of oxidized glutathione (GSSG) to reduced glutathione (GSH), elevate intracellular reactive oxygen species (ROS) and malondialdehyde (MDA) levels, inhibit superoxide dismutase (SOD) activity, induce oxidative stress, and ultimately trigger apoptosis (172).

Additionally, nickel ions induce excessive production of mitochondrial ROS (mtROS), leading to NLRP3 inflammasome activation. This promotes activation of inflammatory caspase-1, enhances IL-1β secretion, and drives the inflammatory response (50).

#### 2.5.5 Oxidative stress affected by lithium

Lithium ions promote Nrf2 induction in the rat peritoneal cavity following spinal cord injury (SCI), thereby upregulating heme oxygenase-1 (HO-1) production, suppressing reactive oxygen species (ROS) generation, and alleviating oxidative stress. This mechanism inhibits inflammasome activation, reduces expression of inflammatory mediators such as TNF-α, IL-6, and IL-8, and mitigates the inflammatory response occurring during spinal cord injury (173).

Conversely, excessive accumulation of lithium ions in the body leads to hyperlithium conditions that promote inflammation. Lithium impairs the function of antioxidant enzymes including glutathione peroxidase, total superoxide dismutase, total antioxidant capacity, and catalase. It induces ROS production, promotes NF-κB phosphorylation and NLRP3 activation, and leads to IL-1β and TNF-α secretion. This exacerbates inflammatory responses and ultimately induces pyroptosis (174).

#### 2.5.6 Oxidative stress affected by yttrium

Yttrium ion intervention induces iron accumulation in cardiomyocytes following YCl_3_ exposure, leading to upregulation of iron metabolism-related proteins TfR1 (transferrin receptor 1) and DMT1 (divalent metal transporter 1), thereby promoting iron uptake. Iron accumulation triggers oxidative stress, elevating MDA levels while decreasing antioxidant enzyme activity of SOD and GSH-Px, thereby exacerbating lipid peroxidation. Oxidative stress and lipid peroxidation induce inflammatory responses, increasing levels of proinflammatory cytokines IL-1β, IL-6, and TNF-α, which further aggravate myocardial damage (175).

#### 2.5.7 Oxidative stress affected by gold

Kobayashi et al. (176) identified that AuNPs activate Nrf2 through three pathways, by promoting ROS generation, by directly binding to the thiol group on Keap1, and by binding to GSH and causing its depletion. Nrf2 can suppress production of inflammatory mediators such as IL-6 and IL-1β (177). El-Sherbiny et al. reported that AuNPs suppress Keap1 (Kelch-like ECH-associated protein 1) expression, activate the Nrf2 antioxidant pathway to enhance cellular antioxidant capacity, reduce the nephrotoxicity of 5-FU (5-fluorouracil), and enhance its antitumor efficacy (178, 179).

#### 2.5.8 Brief summary of oxidative-stress pathway

Metal regulation of cellular redox state divides into consistent pro-oxidants, consistent antioxidants, and metals whose net effect depends on dose or cofactor context (**Table 1**). Iron, nickel and yttrium act as consistent pro-oxidants: Fe²^+^ drives Fenton chemistry to generate hydroxyl and oxygen radicals that trigger lipid peroxidation and ferroptosis, a mechanism also exploited for antibacterial ROS release from Fe-based implants; Ni²^+^ raises the GSSG/GSH ratio and MDA levels while inhibiting SOD, inducing apoptosis, and separately drives mitochondrial ROS-dependent NLRP3/caspase-1/IL-1β activation; and Y³^+^ promotes cardiomyocyte iron accumulation via TfR1/DMT1 upregulation, elevating MDA and lowering SOD/GSH-Px activity to aggravate myocardial oxidative damage. Zinc and gold instead act as consistent antioxidants: Zn²^+^ stimulates Nrf2 and metallothionein (MT1/MT2) expression and directly shields protein thiols from oxidation, while AuNPs activate the Nrf2-Keap1 axis to enhance antioxidant capacity and reduce drug-induced nephrotoxicity. Copper and lithium are bidirectional. Copper both promotes ROS generation—via p47phox/NOX activation feeding the NLRP3 inflammasome and via cuproptosis-associated DLAT aggregation—and serves as an essential cofactor for the antioxidant enzyme Cu/Zn-SOD, which scavenges superoxide. Lithium’s effect is dose-dependent: at physiological/therapeutic levels it promotes Nrf2-HO-1-mediated ROS suppression, whereas excessive lithium impairs antioxidant enzyme capacity and increases ROS, promoting NLRP3-driven pyroptosis. Overall, whether a metal acts as an oxidant or an antioxidant in a given system depends on its accessible oxidation state, its role as an enzymatic cofactor versus a free catalytic ion, and its concentration relative to endogenous antioxidant capacity.

**Table 1:** Overview of regulatory and mechanisms of metals on major inflammatory signaling pathways.

Metal	Pathway	Regulatory direction	Brief effect/mechanism
Fe	NF-κB	Activate	Fe overload activates TLR4/MyD88-p65, raising TNF-α/IL-1β/IL-6; Fe-CpG/IONP constructs engage TLR7/8-MyD88-NF-κB for vaccine adjuvancy; IONP-driven Fenton ROS further activates NF-κB, synergizing with STING agonists
	MAPK	Activate	Fe_3_O_4_ nanoparticles context-dependently up- or down-regulate ERK/JNK/p38; IONPs typically activate this axis, driving M1 polarization and TNF-α/IL-1β/iNOS release
	AKT	Activate	Fe^2+^ is a PHD2 cofactor that normally limits Akt via pVHL/PP2A; iron overload/hypoxia over-activates Akt, promoting thrombosis/inflammation; Fe-oxide nanoparticles can instead inhibit AKT-mTOR, inducing ferroptosis/apoptosis
	STAT	Bidirectional	Iron amplifies IL-6R/JAK1/STAT3, raising IL-6/TNF-α; iron chelation or FAC instead suppresses STAT-driven IL-1β/iNOS/TNF-α
	ROS/Ferroptosis	Activate	Fe^2+^ drives Fenton chemistry, generating hydroxyl/oxygen radicals that cause lipid peroxidation/ferroptosis; Fe-releasing implants exploit this for antibacterial ROS
Mn	NF-κB	Bidirectional	Mn^2+^ activates cGAS-STING-TBK1-p65, raising TNF-α/IL-6/IL-1β/IL-12 and M1 repolarization; Mn-nanomaterials also engage TLR4-MyD88/TRIF; conversely MnO_x_/MnO_2_ nanozymes scavenge ROS to block TLR-NF-κB, and Mn-GCNPs restore MnSOD to inhibit it
Zn	NF-κB	Bidirectional	Zn^2+^ homeostasis (ZIP/ZNT/MT) inhibits IKKβ/IκBα degradation and induces A20, suppressing NF-κB/IL-6; conversely Zn-nanosheets activate Kv1.3-Ca^2+^-calmodulin-NF-κB, raising IL-1β
	MAPK	Inhibit	Zn^2+^ downregulates p-JNK/p-ERK (sparing p38), reducing apoptotic signaling; Zn-releasing scaffolds instead activate p38 to promote bone regeneration/angiogenesis
	AKT	Activate	Zn^2+^ inhibits PP2A, activates Ras/PTEN-degradation and IGF-1R inputs to sustain Akt phosphorylation; ZnO nanoparticles promote AKT methylation, improving intestinal barrier in colitis
	STAT	Inhibit	Zn^2+^ homeostasis inhibits JAK1/STAT3 (↓IL-1β/IL-6/IGF-1) while promoting STAT6 (anti-inflammatory IL-4) and JAK1/STAT1/MMP-10 for bone repair
	Oxidative stress	Inhibit	Zn^2+^ induces Nrf2/metallothionein (MT1/MT2), suppressing ROS and protecting protein thiols
Cu	NF-κB	Bidirectional	Cu^2+^-induced ROS activates TLR4-MyD88-IRAK-TRAF6-TAK1-IKK-NF-κB, raising IL-6/IL-1β/TNF-α/NLRP3; conversely CuO nanozymes/Cu-peptides scavenge ROS or block IκBα phosphorylation, suppressing NF-κB and aiding wound healing
	MAPK	Activate	Cu^2+^ binds MEK1 to activate ERK1/2, promoting tumor growth, and drives ROS-dependent p38/ERK causing neuronal damage; CuO nanoparticles activate JNK/p38/ERK, worsening tissue injury; chelation reverses this
	STAT	Inhibit	Copper complexes downregulate IFNAR1/IL6ST/PDGFRA and upregulate SOCS, inhibiting STAT2/3 and inducing apoptosis; conversely HCC-associated AOC1 activates JAK2-STAT3 for proliferation
	ROS/Cuproptosis	Activate	Excess Cu^2+^ binds DLAT, causing protein aggregation/Fe-S cluster loss and cuproptosis, and activates NOX/NLRP3-caspase-1-IL-1β/IL-18 for M1 polarization; Cu is also an essential Cu/Zn-SOD cofactor for ROS clearance
Mg	NF-κB	Inhibit	Mg^2+^ activates PI3K/AKT to inhibit NF-κB, suppresses TLR4/HMGB1 signaling, and antagonizes Ca^2+^ to lower IL-1β/IL-6/TNF-α; Mg-alloy implants shift the TLR-NF-κB axis toward M2 polarization
	AKT	Activate	Mg^2+^ activates TRPM7-PI3K-AKT, promoting growth; Mg hydride instead suppresses AKT/mTOR-NF-κB/NLRP3 to lower IL-1β; Mg/chitosan hydrogels activate PI3K/AKT to reduce TNF-α/IL-1
Co	NF-κB	Activate	Co^2+^ engages TLR4/MD2 to activate NF-κB, raising TNF-α/IL-8 and CCL20/CXCL10-driven neutrophil chemotaxis; Co-Mn nanoparticles instead reduce ROS/NF-κB, alleviating pulmonary fibrosis
	AKT	Activate	Co^2+^ stabilizes HIF-1α, promoting PI3K/AKT activation and proliferation; inhaled cobalt dust drives PI3K/AKT-NOX4, worsening oxidative stress/carcinogenesis
Ni	NF-κB	Activate	Ni^2+^ promotes IκBα phosphorylation, raising IL-1β/IL-6/IL-8/TNF-α in kidney/liver; nano-NiO activates NIK/IKK-α while downregulating IκBα, causing hepatotoxicity
	MAPK	Activate	Ni^2+^ promotes p38/JNK phosphorylation, raising IL-6/TNF-α and suppressing TGF-β; NiCl_2_/NiS activates ERK-AP-1, inducing TNF-α, Gal-1 and PD-L1
	AKT	Bidirectional	Ni^2+^ inhibits PI3K/AKT/mTOR, promoting autophagy/renal injury; conversely in pulmonary fibroblasts it activates AKT, raising IL-1β/TGF-β/TNF-α and fibrosis
	STAT	Activate	Ni^2+^ induces IL-6/STAT3 phosphorylation, promoting TRIM31-driven cancer invasion, and activates dendritic-cell JAK-STAT to raise IL-27/IL-22, driving allergic contact dermatitis
	Oxidative stress	Activate	Ni^2+^ raises GSSG/GSH ratio, ROS and MDA while inhibiting SOD, inducing apoptosis and mtROS-driven NLRP3/caspase-1/IL-1β
Li	NF-κB	Bidirectional	Low-dose Li^+^ upregulates p50/IκBα and p50 homodimerization, inhibiting NF-κB; overload instead impairs antioxidant enzymes, raising ROS, NF-κB activation and NLRP3-driven IL-1β/TNF-α/pyroptosis
	AKT	Activate	Li^+^ activates PI3K/AKT/mTOR, alleviating glucocorticoid-induced chondrocyte autophagy, and inhibits GSK-3β to promote WNT/β-catenin, lowering NF-κB-driven IL-6/IL-8/TNF-α
	STAT	Inhibit	Li^+^ inhibits GSK-3β, suppressing STAT1 phosphorylation and Th1-driven IFN-γ/TNF-α/IL-2 secretion
	Oxidative stress	Bidirectional	Low-dose Li^+^ promotes Nrf2/HO-1, suppressing ROS/inflammasome activity after spinal cord injury; excess Li^+^ impairs antioxidant enzymes, raising ROS and pyroptosis
Ag	NF-κB	Bidirectional	AgNPs promote IκBα degradation, activating NF-κB-COX2 and IL-6/IL-8/IL-1α; biogenic (plant-derived) AgNPs instead inhibit TLR4/MyD88-NF-κB, reducing inflammation
Au	NF-κB	Inhibit	Peptide-coated Au nanoclusters/nanoparticles inhibit LPS-induced NF-κB, suppressing TNF-α/IL-1β/IL-6/iNOS and promoting protective M2 polarization
	Oxidative stress	Inhibit	AuNPs activate Nrf2 via ROS-mediated Keap1 thiol modification/GSH depletion, boosting antioxidant capacity and reducing 5-FU nephrotoxicity
La	NF-κB	Bidirectional	La^3+^ stimulates macrophage NF-κB (↑IL-1β/IL-8) and blocks STING degradation to boost antitumor immunity; conversely it inhibits PKC/Ca^2+^-IκBα-NF-κB (↓NO/TNF-α) and RANK/RANKL-NF-κB (bone protection); La_2_O_3_ nanoparticles induce NF-κB-p65-driven apoptosis/reproductive toxicity
	MAPK	Inhibit	La^3+^ inhibits JNK2/p38 (sparing ERK1/2), lowering inflammation and improving atherosclerosis via ↓ICAM-1/MCP-1/NF-κB-p65; conversely it can activate ERK/JNK to inhibit vascular calcification
Ce	NF-κB	Bidirectional	High-dose Ce^3+^/CdTe QDs activate Nox1/RANKL-NF-κB, promoting osteoclastogenesis and TNF-α/IL-1β/IL-6; conversely cerium oxide nanoparticles (CONPs) scavenge ROS and shift miR-155↓/miR-124,146a↑ to suppress NF-κB/NLRP3/caspase-1-driven IL-1β/IL-18
Y	Oxidative stress	Activate	YCl_3_ induces iron accumulation by upregulating TfR1 and DMT1, promoting iron uptake; this triggers oxidative stress, elevates MDA, reduces SOD/GSH-Px activity, and increases IL-1β, IL-6, and TNF-α, aggravating myocardial inflammation and damage.

## 3 Metal-based clinical therapies status

Regarding clinical translation, metal-based and metal-nanoparticle immunotherapeutics currently present a stratified landscape comprising agents that are already approved, agents that are actively progressing through clinical trials, and agents that have failed to demonstrate clinical benefit. Among approved or clinically applied formulations, the superparamagnetic iron oxide nanoparticle preparation NanoTherm^®^ (MagForce) received CE certification in the European Union in 2010 as a Class III medical device for magnetic thermal ablation of recurrent glioblastoma; a prospective phase II study combining this therapy with fractionated stereotactic radiotherapy reported a median overall survival from first recurrence (OS-2) of 13.4 months—substantially exceeding historical controls—generally manageable safety profile; the most common events were sweating, localized warmth, and transient headache, although infrequent neurological events such as convulsions were also noted (180). In the gold nanoparticle domain, the PEGylated colloidal gold–TNF-α conjugate Aurimune (CYT-6091) demonstrated tumor-targeted accumulation and a favorable safety profile, enabling the administration of TNF-α doses that were otherwise poorly tolerated as a free agent (181, 182), while a 150-nm silica–gold nanoshell formulation analogous to AuroLase^®^ has been evaluated in prospective randomized controlled trials for photothermal treatment of moderate-to-severe inflammatory acne vulgaris, showing good local tolerability with only mild reddening and swelling (183).

Among representative agents currently advancing through clinical trials, Mn2+, acting as a direct activator of the cGAS-STING pathway, was evaluated in a first-in-human, dose-escalation phase I trial (NCT03991559) in combination with anti-PD-1 antibody therapy in 22 patients with advanced metastatic solid tumors or lymphomas who had received at least two prior lines of systemic therapy; this yielded a best objective response rate of 45.5% and a disease control rate of 90.9% without severe toxicity, and has since progressed to phase II evaluation (184). A single-center, single-arm trial of biodegradable magnesium-based embolic microspheres (BM601) combined with transarterial chemoembolization (TACE) in hepatocellular carcinoma reported an objective response rate of 93.3%, with 11 of 15 patients (73.3%) achieving complete response, suggesting that magnesium-based degradation products may indirectly potentiate antitumor immunity by neutralizing the acidic tumor microenvironment and alleviating hypoxia (185). Additionally, a phase I trial of AuroLase^®^-mediated photothermal therapy for refractory/recurrent head and neck cancer has been completed, with a follow-up trial evaluating its efficacy against primary or metastatic lung tumors causing airway obstruction currently underway (186); the feasibility and safety of magnetic nanoparticle-mediated thermotherapy have likewise been demonstrated in an early-phase trial for locally recurrent prostate cancer (187). Gold nanoparticles conjugated with nucleic acid therapeutics (NU-0129) are also being evaluated in an ongoing trial (NCT03020017) for gliosarcoma and recurrent glioblastoma, reflecting continued diversification of metal-nanoparticle platforms entering clinical evaluation (188).

By contrast, disulfiram (DSF), an aldehyde dehydrogenase inhibitor that promotes intracellular copper accumulation, illustrates the challenges of clinical translation despite strong preclinical rationale: although DSF/copper combinations showed synergistic antitumor activity with agents such as temozolomide in multiple preclinical models, a randomized phase II/III trial in 88 patients with recurrent glioblastoma (NCT02678975) found that DSF plus copper added to standard alkylating chemotherapy failed to improve overall survival and was associated with increased toxicity, missing its intended efficacy endpoint (189). More broadly, the clinical translation of nanomedicines is also constrained by underdeveloped regulatory standards and insufficient long-term biosafety data, factors that have contributed to the discontinuation of a number of nanoparticle-based clinical studies and that similarly bear on the trajectory of metal-based immunotherapeutic candidates (190).

## 4 Perspective

Metallic elements exhibit diverse and bidirectional mechanisms in regulating inflammatory responses. This systematic review examines how various metals—including iron, zinc, copper, magnesium, cobalt, nickel, silver, lithium, gold, lanthanum, and cerium—modulate inflammation through signaling pathways such as NF-κB, MAPK, AKT, STAT, and ROS-related pathways.

In the NF-κB pathway, iron overload promotes NF-κB phosphorylation by activating the TLR4/MyD88 axis, thereby exacerbating inflammation. Conversely, zinc exerts anti-inflammatory effects by inhibiting IKKβ or binding to the A20 protein, thereby blocking pathway activation. Copper and magnesium may also attenuate inflammation by inhibiting IκBα phosphorylation or TLR4 expression. However, at high concentrations, copper and nickel conversely activate NF-κB, enhancing inflammatory responses. Within the MAPK pathway, iron and copper promote inflammatory cytokine release by activating kinases such as ERK, JNK, and p38, whereas zinc intervenes in inflammation by regulating matrix metalloproteinase (MMP) activity. In the AKT pathway, zinc and magnesium exert anti-inflammatory effects by activating PI3K/AKT signaling; conversely, nickel inhibits this pathway, inducing cellular damage or autophagy. Within the STAT pathway, iron suppresses STAT1 activation, while zinc promotes STAT6 phosphorylation—both contributing to inflammation mitigation; nickel exacerbates inflammation by activating STAT3. Furthermore, ROS, a key inflammatory mediator, undergoes precise regulation by metallic elements. Iron promotes ROS generation via the Fenton reaction, intensifying oxidative stress. Copper also modulates the immune microenvironment through the cuproptosis pathway.

While these intricate signaling pathways underscore the fundamental regulatory roles of metal ions, the net immunomodulatory outcomes in biological systems are frequently complex, context-dependent, or even paradoxical. To fundamentally resolve the reported ‘bidirectional’ or paradoxical immunomodulatory effects of metals, it is critical to dissect formulation-, valence-dependent variables from intrinsic ion biology. The intracellular fate of gold nanoparticles (AuNPs) is strictly dictated by endocytic pathways; particles entering lysosomes undergo acidic dissolution to release Au+ ions that trigger mitochondrial dysfunction and apoptosis via thioredoxin reductase inhibition, whereas direct membrane-penetrating particles bypass ion release. Similarly, silver nanoparticles (AgNPs) release Ag+ in acidic endolysosomes to induce oxidative stress, while free Ag+ directly surges ROS levels—a toxicity effectively blunted by ion chelators (191). Hexavalent chromium utilizes sulfate transporters to enter cells, generating free radicals during intracellular reduction to drive DNA adducts and mutagenesis, whereas trivalent chromium exhibits minimal passive uptake and lower toxicity (192). For copper, Cu2+ must be reduced to Cu+ by FDX1 to exert cuproptosis, where Cu+ directly binds lipoylated proteins to induce toxic protein aggregation and iron-sulfur cluster loss, distinct from apoptosis or ferroptosis (152).

In the future, multi-metal alloys will become the core direction for metal immunotherapy. By precisely designing synergistic mechanisms between metals, novel alloy medical materials with multifunctional responsiveness are ushering in an intelligent era of transition from “single-metal intervention” to “multi-metal coordinated regulation.” Research has demonstrated that hyaluronic acid-modified zinc-iron bimetallic peroxide nanoparticles (Fe-ZnO_2_@HA) can simultaneously trigger pyroptosis and ferroptosis within the tumor microenvironment, reshape the immunosuppressive matrix, and significantly enhance the antitumor efficacy of the immune checkpoint inhibitor αPD-1 (193). Additionally, Gd-MOF-5 synergistically downregulates immunosuppressive signals (phosphatidylserine, IL-10, TGF-β) and upregulates immune-activating signals (calreticulin, IL-12, TNF-α) by inhibiting phosphatidylserine efflux via Gd³^+^ and inducing immunogenic cell death through Zn²^+^. This enhances antitumor immune responses and boosts the efficacy of immune checkpoint inhibitors (αPD-L1) (194). Multimetallic alloys can simultaneously enhance targeting efficiency, improve drug safety, and enable adjustable controlled release timing in applications such as implant materials, delivery carriers, and multimetallic MOF particles. This provides a scalable, reusable technological platform for the clinical translation of metal immunotherapy.

However, given the vast compositional and structural space of multimetallic systems, it is particularly crucial to move away from traditional trial-and-error experiments toward a computational and data-driven rational design approach. Taking nanoenzymes as an example, their rational design increasingly relies on density functional theory (DFT) calculations and first-principles simulations to elucidate elementary reaction pathways and atomic-scale mechanisms. Establishing universal activity descriptors—such as adsorption energy, active site geometry, or electronic configuration—can provide clear guidance for material design and optimization. Furthermore, combining machine learning (ML) with computational databases enables high-throughput screening prior to synthesis and the predictive modeling of complex “structure-property” relationships. Extending such computational frameworks to a broader range of nanomaterial systems will significantly narrow the gap between theoretical predictions and targeted functional customization (195).

Future efforts should also integrate materials genomics with high-precision quantification methods to predict the immunomodulatory properties of metals and alloys. This should be complemented by systematically consolidating experimental data to collect activation levels of inflammatory cytokines (IL-6, IL-8, TNF-α, IL-10), chemokines (IL-8, MCP-1), enzymes (COX-2, iNOS, MMP-2, caspase-1), and autophagy-related molecules (Atg5, Beclin1, mTOR). These parameters should be fitted to nanoparticle intake doses and implant metal exposure times to derive relevant curves, thereby refining the causal data chain linking “dose-exposure-immunological readouts-clinical endpoints” and establishing a specialized metal immunology database. Further data mining and modeling through machine learning will guide rational design and performance optimization of metallic materials and composite systems for immunotherapy, enabling the development of more precise novel metallic therapeutics (**Figure 10**).

**Figure 10:**
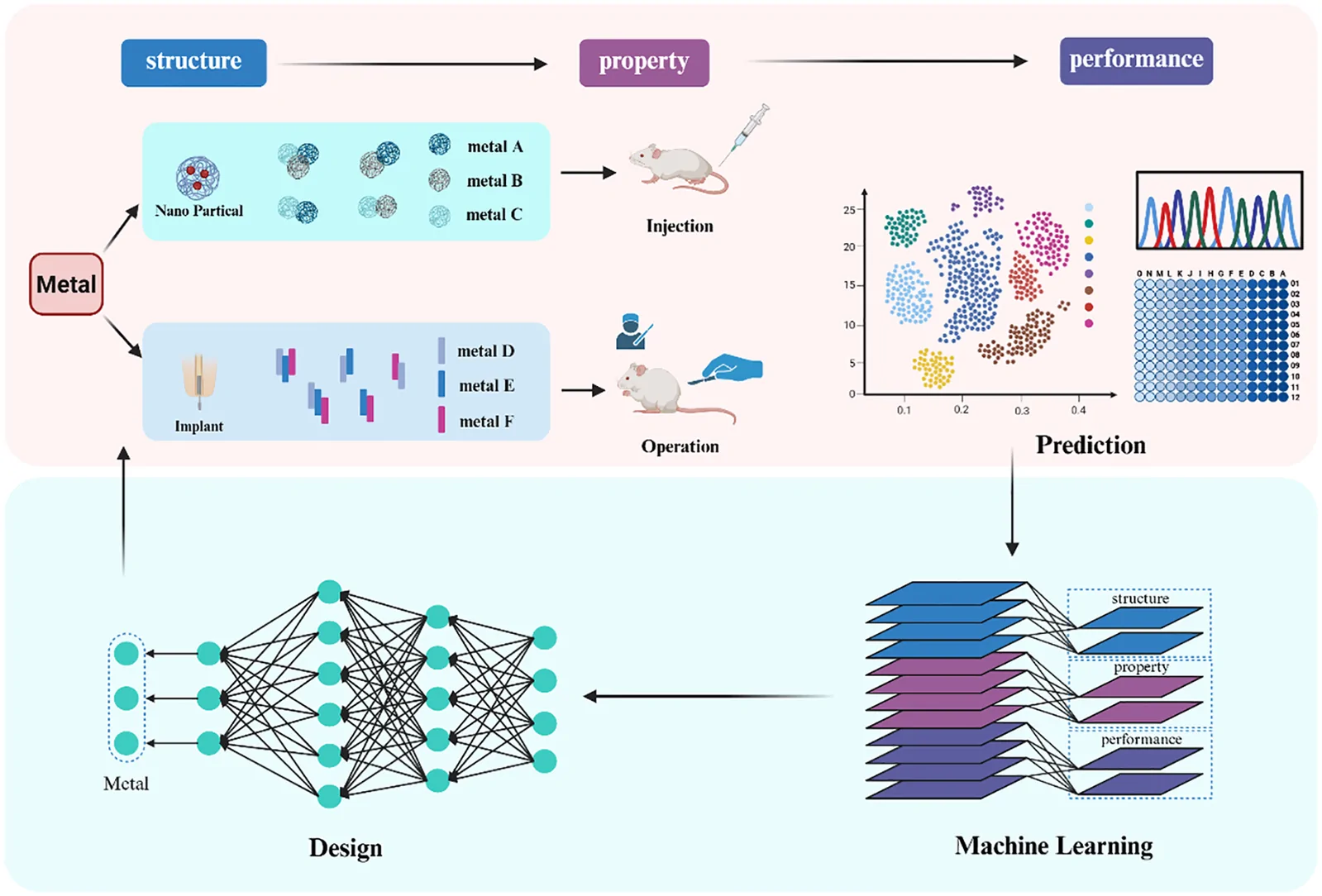
The perspective of metal materials genomics.
